# Vitamin A treatment restores vision failures arising from Leber’s hereditary optic neuropathy–linked mtDNA mutation

**DOI:** 10.1172/jci.insight.188962

**Published:** 2025-03-04

**Authors:** Cheng Ai, Huiying Li, Chunyan Wang, Yanchun Ji, Douglas C. Wallace, Junbin Qian, Yimin Zhu, Min-Xin Guan

**Affiliations:** 1 Center for Mitochondrial Biomedicine and Department of Ophthalmology, the Fourth Affiliated Hospital, Zhejiang University School of Medicine, Yiwu, China.; 2 Institute of Genetics, Zhejiang University, Hangzhou, China.; 3 Center for Genetic Medicine, Zhejiang University International School and Institute of Medicine, Yiwu, China.; 4 Zhejiang Key Laboratory of Precision Diagnosis and Therapy for Major Gynecological Diseases, Women’s Hospital, Zhejiang University School of Medicine, Hangzhou, China.; 5 Division of Medical Genetics and Genomics, The Children’s Hospital, Zhejiang University School of Medicine and National Clinical Research Center for Child Health, Hangzhou, China.; 6 Center for Mitochondrial and Epigenomic Medicine, Children’s Hospital of Philadelphia, Philadelphia, Pennsylvania, USA.; 7 Zhejiang Provincial Key Laboratory of Genetic and Developmental Disorders, Zhejiang University, Hangzhou, China.

**Keywords:** Genetics, Metabolism, Ophthalmology, Genetic diseases, Mitochondria, Mouse models

## Abstract

Leber hereditary optic neuropathy (LHON) is a paradigm for mitochondrial retinopathy due to mitochondrial DNA (mtDNA) mutations. However, the mechanism underlying retinal cell–specific effects of LHON-linked mtDNA mutations remains poorly understood, and there has been no effective treatment or cure for this disorder. Using a mouse model bearing an LHON-linked ND6^P25L^ mutation, we demonstrated that the mutation caused retinal cell–specific deficiencies, especially in retinal ganglion cells (RGCs), rods, and Müller cells. Single-cell RNA sequencing revealed cell-specific dysregulation of oxidative phosphorylation and visual signaling pathways in the mutant retina. Strikingly, ND6 mutation–induced dysfunctions caused abnormal vitamin A (VA) metabolism essential for visual function. VA supplementation remarkably alleviated retinal deficiencies, including reduced fundus lesion and retinal thickness and increased numbers of RGCs, photoreceptors, and Müller cell neurites. The restoration of visual functions with VA treatment were further evidenced by correcting dysregulations of phototransduction cascade and neurotransmitter transmission and restoring electrophysiological properties. Interestingly, VA supplementation markedly rescued the abnormal mitochondrial morphologies and functions in the mutant retina. These findings provide insight into retina-specific pathophysiology of mitochondrial retinopathy arising from VA deficiency and mitochondrial dysfunction induced by mtDNA mutation and a step toward therapeutic intervention for LHON and other mitochondrial retinopathies.

## Abstract 2

The LHON-linked mtDNA mutation caused the deficient vitamin A metabolism and the administration of vitamin A rescued the retinal deficiency arising from LHON-associated mtDNA mutation.

## Graphical abstract

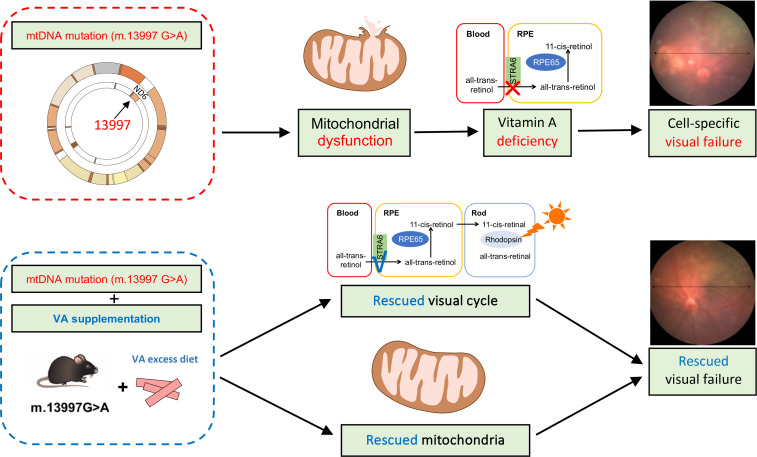

## Introduction

Human retina is the high-energy-demanding tissue that is composed of 5 major neuronal cell classes — photoreceptors, bipolar cells, retinal ganglion cells (RGCs), horizontal cells, and amacrine cells — and Müller cells (1–3). Healthy mitochondria to produce cellular energy through oxidative phosphorylation (OXPHOS) are important for retinal cell function and normal vision (4–6). The pigmented/photoreceptor layers and ganglion cell/nerve fiber layers of retina are exquisitely vulnerable to OXPHOS impairment (6–10). These mitochondrial dysfunctions lead to retinal dystrophy, including retinitis pigmentosa and optic atrophy (5, 6). Leber hereditary optic neuropathy (LHON) is a paradigm for mitochondrial retinopathy, characterized by bilateral, painless, subacute, central visual loss in young adults (11–14). Retinal deficiencies in this disorder include the primary degeneration of RGCs accompanied by decreased thickness of retinal nerve fiber layer (13, 14). Mutations in mitochondrial DNA (mtDNA) have been identified as contributing to the pathogenesis of LHON to various extents (11–15). These LHON-associated mtDNA mutations, including NADH dehydrogenase subunit 1 gene (ND1) 3460G>A (A52T), ND4 11778G>A (R340H), ND6 14484T>C (M64V), and ND6 14600G>A (P25L) mutations, primarily affect the essential subunits of complex I (NADH: ubiquinone oxidoreductase) (11–19). LHON-associated mtDNA mutation–induced complex I dysfunctions impair the synthesis of ATP, increase generation of reactive oxygen species (ROS), and prompt apoptosis (20–26). However, the mechanism underlying the tissue-/cell-specific manifestation of LHON-associated mtDNA mutations is far from understood, and there is no effective treatment or cure for this disease.

A mouse bearing the LHON-linked ND6 P25L mutation, which exhibits RGC degeneration and preferential loss of the smallest fibers, is an excellent disease model for elucidating the pathophysiology of this disease, specifically the tissue-specific manifestations, and developing the therapy approach (27). In this study, using this disease model, we investigated the mechanism underlying retinal cell–specific manifestation of ND6^P25L^ mutation and therapeutic intervention for this disease. The mutant mice were assessed for the retinal cell–specific manifestations, including RGCs, other neuronal cell types, and Müller cells. To obtain a detailed single-cell transcriptome of the retina, we applied single-cell RNA sequencing (scRNA-Seq) technology to this mouse model. Based on scRNA-Seq data, we examined the cell-specific effects of mitochondrial dysfunctions, including the expression of genes involved in OXPHOS biogenesis and mitochondrial morphology and functions on the mouse retina. We then evaluated the impact of ND6 mutation on visual signaling pathways and retinal functions. Strikingly, scRNA-Seq analysis of metabolic profiles revealed the dysregulation of vitamin A (VA) (retinol) metabolism in the mutant retina. Indeed, VA and its derivatives, such as retinoic acid, play important roles in the eye development and visual function, particularly by regenerating the visual chromophore of rhodopsin for light reception (28–30). Furthermore, retinoic acid exerts an effect on the OXPHOS, mitochondria biogenesis, and oxidative stress (31–33). The VA deficiency often causes retinal diseases, including retinal pigment epithelium–related (RPE-related) disorders, and VA palmitate supplement improves vision in patients with retinitis pigmentosa (28, 34). Hence, the VA administration may act as one therapeutic approach to restore vision failures arising from LHON-linked mtDNA mutation. We then investigated if that VA supplementation improved deficient VA metabolism, rescued mitochondrial morphology and function, and subsequently restored the retinal deficiencies due to LHON-linked ND6 mutation in the ND6^P25L^ mutant and wild-type (WT) mice.

## Results

### scRNA-Seq reveals changes in retinal composition and transcriptional profiles.

**Figure 1:**
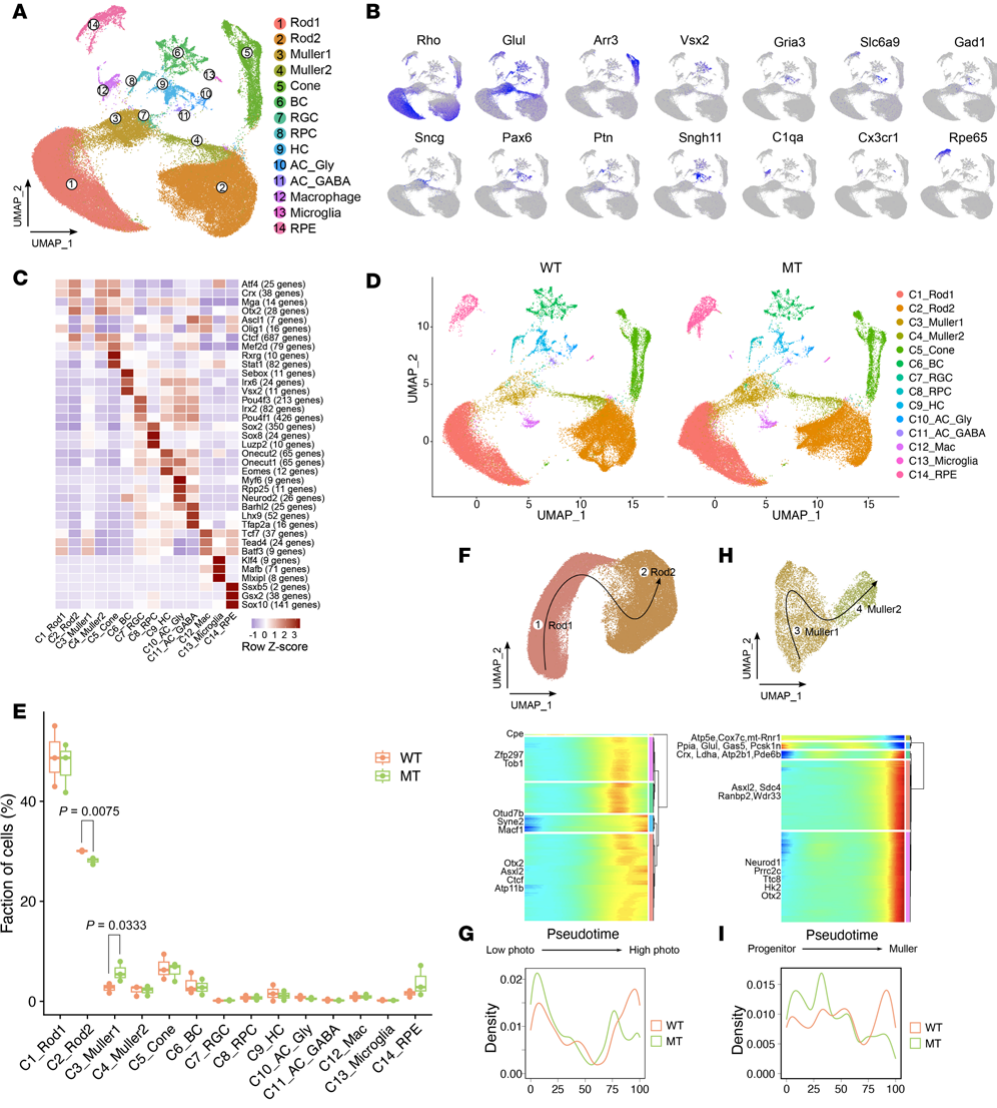
Mouse retinal compositional and transcriptional profiles. (**A**) Uniform manifold approximation and projection (UMAP) map of 83,672 merged retinal cells from WT and mutant (MT) mice color coded for the indicated cell type. BC, bipolar cell; RPC, retina progenitor cell; HC, horizontal cell; AC_Gly, glycinergic amacrine cell; AC_GABA, GABAergic amacrine cell. (**B**) UMAP plots showing the expression of marker genes for each cell type. (**C**) The expression of transcription factors for each cell type. (**D**) UMAP plots of retina cells from WT (left) and MT (right) mice. (**E**) Box plots showing the fractions of cells for each cell type in each mouse. Box plots show the interquartile range, median (line), and minimum and maximum (whiskers). (**F** and **H**) Pseudotime trajectories of Rod1, Rod2 (**F**), Muller1, and Muller2 (**H**) based on Slingshot and gene expression dynamics along the trajectory. Genes clustered into 5 gene sets, each characterized by specific expression profiles, as depicted by a selection of marker genes characteristic for each cluster. (**G** and **I**) Plots showing cell density of Rod1, Rod2 (**G**), Muller1, and Muller2 (**I**) along the trajectory comparing WT and MT.

To investigate the impact of the ND6 mutation on retinal cell composition and transcriptional profiles, we conducted droplet-based scRNA-Seq on retinal cells from 7-month-old ND6^P25L^ and WT mice. These analyses produced high-quality profiles for 83,672 cells. Following quality filtering, we integrated these profiles using canonical correlation analysis, performed differential expression analysis, and categorized the cells into 14 distinct clusters based on known gene markers (Figure 1, A and B, and Supplemental Figure 1A; supplemental material available online with this article; https://doi.org/10.1172/jci.insight.188962DS1) (35–37). These markers included *Arr3*^+^ (cones), *Vsx2*^+^ (bipolar cells), *Sncg*^+^ (RGCs), *Ptn*^+^ (retinal progenitor cells), *Snhg11*^+^ (horizontal cells), *Gria3*^+^ (amacrine cells), *Slc6a9*^+^ (AC_Gly), *Gad1*^+^ (AC_GABA), *C1qa*^+^ (macrophages), *Rpe65*^+^ (RPE cells), *Glul*^+^ (Müller cells), and *Rho*^+^ (rods). Transcription factor activities in cell clusters were verified using SCENIC (Figure 1C) (38).

To examine if the ND6 mutation affected retinal subset composition, we analyzed the relative proportions of each cell cluster among mutant and WT mouse retina. Figure 1, D and E, summarize marked variations in the cell type composition over 14 cell clusters between mutant and WT mouse retina. The mutant retina exhibited various reductions in neuronal cell types, including cones, bipolar cells, horizontal cells, and Muller2 cells, with especially pronounced effects on Rod2 cells, but significantly increased proportions of Muller1 cells, as compared with those in WT retina.

The transcriptomic changes in the rod and Müller cells were further evaluated using pseudotime trajectory analysis with the Slingshot package (Figure 1, F–I). The mutant retinas revealed a higher density of Rod1 and Muller1 cells but a lower density of Rod2 and Muller2 cells, as compared with WT retinas (Figure 1, G and I). Rod2 cells exhibited higher expression of photoreceptor differentiation transcription factors, such as *Otx2* and *Crx*, but lower expression of OXPHOS markers, including ND4 and CYTB, than in Rod1 cells, indicating that Rod2 cells may be more differentiated and have specific mitochondrial features (Figure 1F and Supplemental Figure 1, B and C) (39–41). Moreover, Muller1 cells expressed higher levels of progenitor transcription factors *Ascl1* and *Olig1*, but lower levels of *Otx2* and *Crx*, than in Muller2 cells, suggesting that Muller1 cells may function as the progenitors for major retinal cell types (Figure 1H and Supplemental Figure 1, D and E) (42, 43).

### Retinal cell–specific defects in transcriptional regulation of OXPHOS pathways.

**Figure 2:**
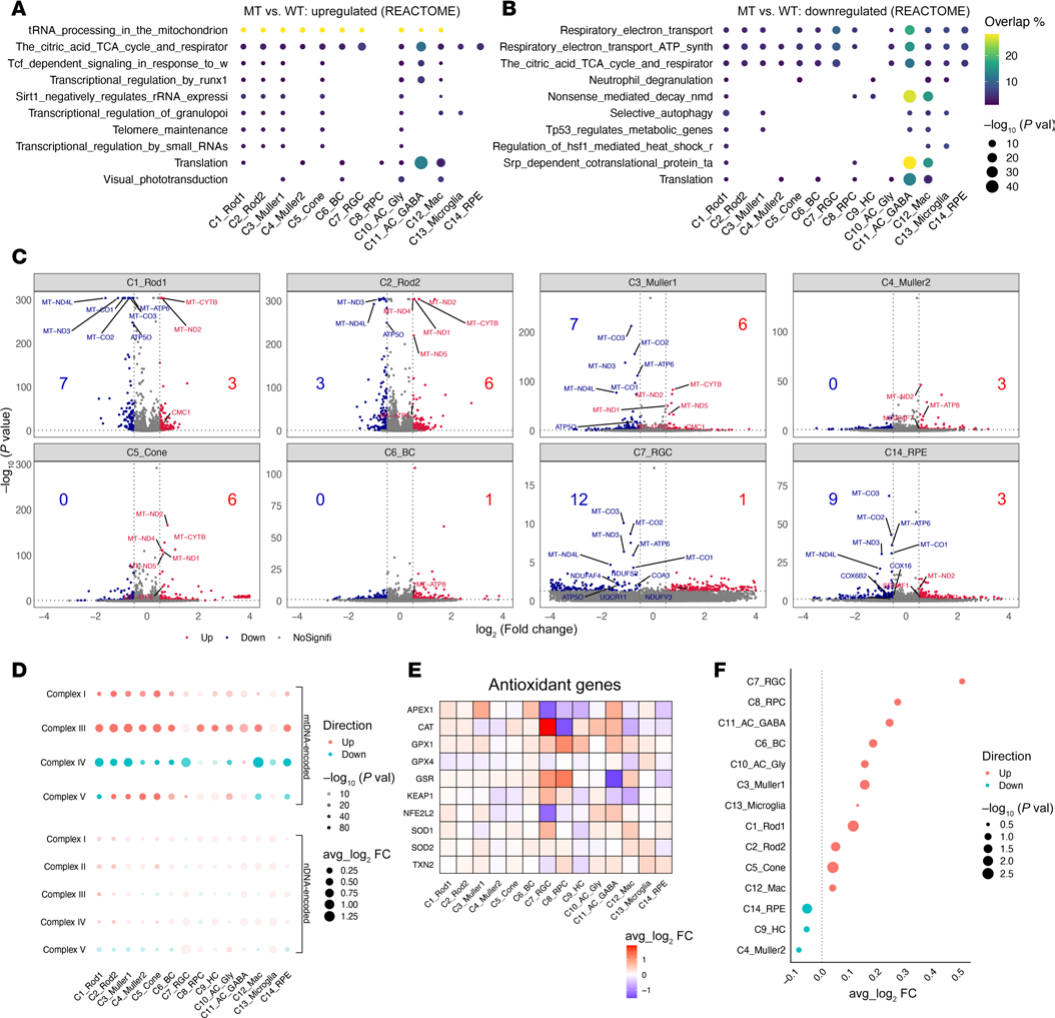
Cell-specific mitochondrial dysfunctions in the retina. (**A**) Dot plots showing the significance (−log_10_ *P* value) and overlap (percentage of differentially expressed genes; DEGs) of upregulated REACTOME pathways in the majority of cell clusters in MT retina by gene set enrichment analysis on DEGs, as compared with WT. (**B**) Dot plots showing the significance (–log_10_ *P* value) and overlap of downregulated REACTOME pathways in the majority of cell clusters in MT retina, as compared with WT. (**C**) Volcano plots showing DEGs comparing MT and WT mice in 8 retina subsets (Supplemental Figure 2A shows the remaining 6 retina subsets). Dots on the volcano plot: gray, no significant change; red, *P* < 0.05 and log_2_FC ≥ 0.5 (FC, fold change); blue, *P* < 0.05 and log_2_FC ≤ –0.5. The significantly differentially expressed OXPHOS genes were labeled. The counts of significantly up- and downregulated OXPHOS genes for each cluster were labeled. (**D**) Dot plots showing the average fold-change of expression of mtDNA-encoded and nDNA-encoded OXPHOS genes in each OXPHOS complex across retina subsets, comparing MT and WT mice. (**E** and **F**) Heatmap displaying expression of genes involved in the antioxidant pathway across subsets (**E**) and dot plot showing the quantification of average expression levels of the antioxidant pathway across subsets (**F**).

We performed differential expression analysis and scored REACTOME pathway activity for each cell subset in the mutant and WT retinas. The pathway analysis revealed the dysregulation of OXPHOS pathways among the majority of cell clusters in mutant retina. In particular, upregulated pathways included mitochondrial transfer RNA (tRNA) processing, tricarboxylic acid (TCA) cycle, and transcriptional regulation of small RNAs, while downregulated pathways were primarily involved in OXPHOS biogenesis, such as electron transport chains, ATP synthase, and TCA cycle (Figure 2, A and B). Furthermore, several phototransduction cascade pathways were upregulated in the C3_Müller1, C6_BC, and C10_AC_Gly (Figure 2A), suggesting that these cell types were particularly vulnerable to mitochondrial dysfunctions.

To further assess the effects of ND6 mutation on the OXPHOS pathways, we focused on 152 (13 mtDNA and 139 nuclear DNA [nDNA], MitoCarta3.0 database) genes involved in OXPHOS biogenesis in the differential expression analysis of retinal subsets (44). As shown in Figure 2C and Supplemental Figure 2A, 13 mtDNA-encoded and 38 nDNA-encoded genes in the mutant retina exhibited differential expression across 14 subsets as compared with WT retina (absolute logFC > 0.5 and *P* value < 0.05). Notably, RGCs exhibited many more downregulated genes than upregulated genes. More downregulated than upregulated genes were also observed in Rod1, RPE, and macrophages. Conversely, more upregulated than downregulated genes occurred in the cone, HC, GABAergic AC, Rod2, and Muller2, respectively.

We then examined the expression levels of mtDNA and nDNA genes encoding components for each of the OXPHOS complexes (Supplemental Table 1). Similar expression patterns of mtDNA-encoded subunits, including upregulation of ND2, CYTB, and ATP8 but downregulation of ND3, ND4L, CO1, CO2, CO3, and ATP6, occurred among 14 cell types in the mutant retina (Figure 2D and Supplemental Figure 3). These mRNA expressions were further confirmed by quantitative PCR analysis (Supplemental Figure 2B). By contrast, the expression levels of nDNA-encoded OXPHOS subunits and assembly factors varied greatly across the 14 subsets in the mutant retina. Further expression analysis of genes associated with complex I assembly modules showed only downregulation of the ND2 module, including ND6, but upregulation of other 5 modules (Supplemental Figure 3).

ROS production acts as a major mitochondrial function involved in cellular adaptation and stress resistance (45). Mitochondrial dysfunctions increase the ROS production and therefore regulate the expression of antioxidant pathways (23, 24, 46). We then examined the effects of ND6 mutation on antioxidant pathways in mouse retina. As shown in Figure 2, E and F, 11 clusters, including cones and Rod2, exhibited variable upregulation of antioxidant pathways, such as *SOD2*, with particularly pronounced upregulation in the RGCs, whereas 3 clusters, including RPE, showed downregulation of these pathways. These data indicated that the ND6 mutation caused cell-specific mitochondrial defects in retinal subsets.

### Dysregulation of visual signaling pathways and VA metabolism.

**Figure 3:**
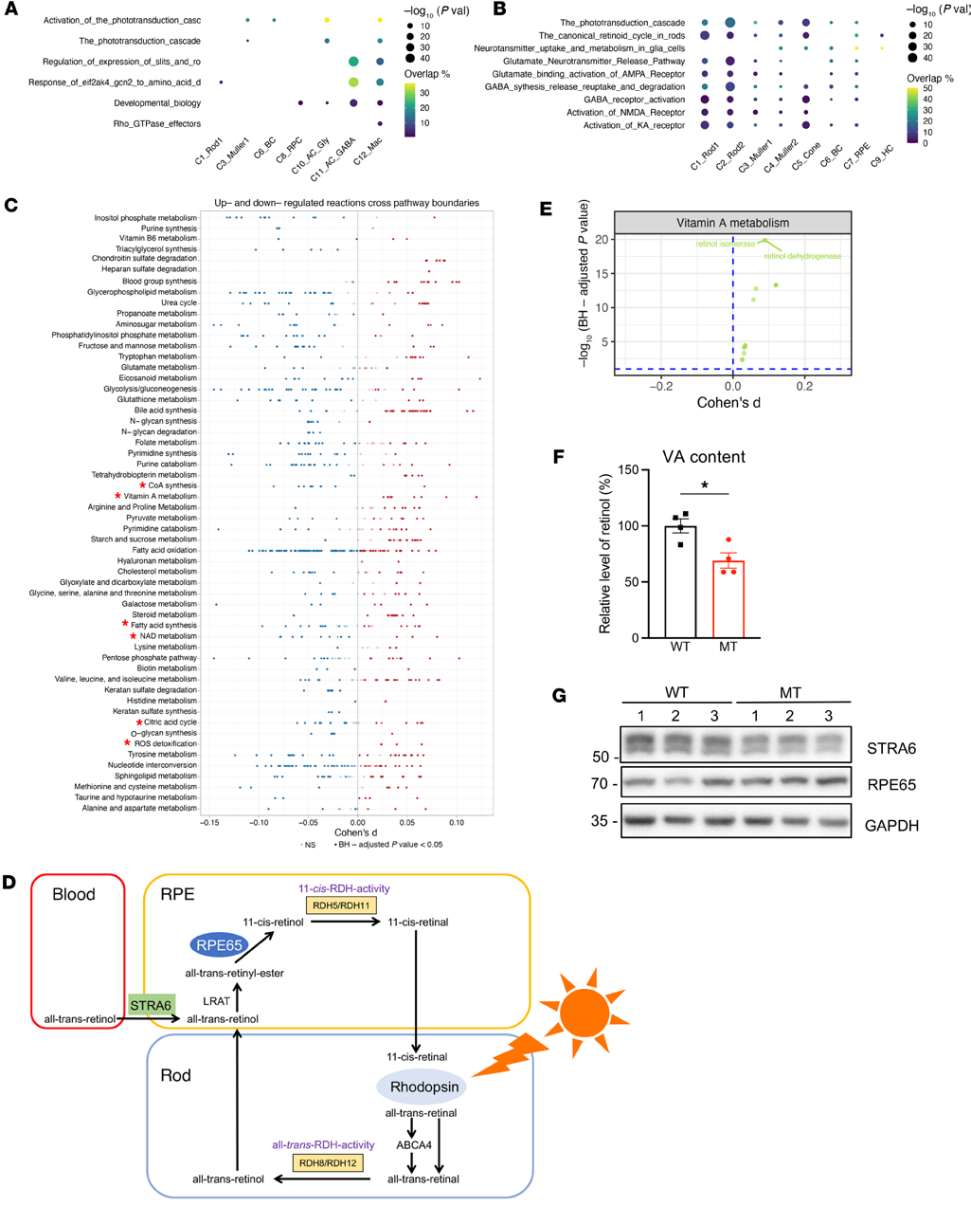
Abnormal visual signaling pathways and VA metabolism. (**A**) Dot plots showing the significance (–log_10_ *P* value) and overlap of differentially expressed cell-specific REACTOME pathways in MT retina, as compared with WT. (**B**) Dot plot showing the significance (−log_10_ *P* value) and overlap of vision-related signaling pathways (rows) in retina clusters (columns) between MT and WT mice. (**C**) Compass-based exploration of metabolic change in MT retina, compared with WT retina. Reactions (dots) are partitioned by Recon2 pathways and colored by the sign of their Cohen’s d statistic. Red asterisks indicate the significantly dysregulated metabolic pathways. BH, Benjamini-Hochberg. (**D**) Schematic illustration of VA metabolism (visual cycle) in retina. (**E**) Compass score differential activity test of VA metabolism pathways between MT and WT retina. Each dot represents a single biochemical reaction. (**F**) Relative levels of retinol in eyeballs of MT and WT mice using ELISA. Data are shown as mean ± SEM. *P < 0.05; *n* = 4 mice per group; 2-tailed unpaired Student’s *t* test. Normality was assessed using the Shapiro-Wilk test (*P* = 0.8986), and equal variance was confirmed using the *F* test (*P* = 0.8697). (**G**) Immunoblot analysis of proteins involved in VA metabolism. Total cellular proteins in WT and MT retina were electrophoresed with PAGE and hybridized with RPE65 and STRA6 antibodies and GAPDH as a loading control, respectively. Values on left represent kilodaltons.

We then assessed the cell-specific responses to mitochondrial dysfunctions caused by the ND6 mutation in the retinal subsets (Figure 3A). Phototransduction cascade and its activation pathways were specifically dysregulated in Müller1, BC, AC_Gly, and macrophages, indicating that visual function is cell-specifically susceptible to mitochondrial defects. To further investigate the consequences of the ND6 mutation on retinal function, we focused on the assessment of expression of vision-related signaling pathways in mutant and WT mice (Supplemental Table 2). As shown in Figure 3B, ND6 mutant retina exhibited marked dysregulation of the phototransduction cascade, canonical retinoid (VA) cycle, and neurotransmission pathways, including neurotransmitter uptake, glutamate release, and GABA release and reuptake.

To systematically evaluate metabolic changes in ND6 mutants, we employed the Compass algorithm for comprehensive single-cell metabolism characterization (47). Using flux balance analysis, we showed significant differences in metabolic profiles, including up- and downregulated reactions across pathway boundaries between mutant and WT retinas (Figure 3C). In particular, the mutant retina exhibited the downregulation of mitochondrial and cytosolic bioenergetic pathways, including glycolysis, CoA synthesis, fatty acid oxidation, NAD metabolism, and TCA cycle, but upregulation of ROS detoxification enzymes (Figure 3C and Supplemental Figure 4).

Strikingly, the VA metabolism was significantly upregulated in the mutant retina (Figure 3C). We then further assessed the impact of ND6 mutation–induced abnormal metabolism of VA on retina function. In fact, VA is delivered from the blood to RPE cells via retinoid binding protein receptor STRA6 (28). It then undergoes a visual cycle in RPE, photoreceptor cells, and Müller cells to regenerate the visual chromophore of rhodopsin for light reception (Figure 3D) (28–30). Compass analysis exhibited significant upregulation of retinol isomerase and reductase in the mutant retina (Figure 3E). Aberrant VA metabolism was further verified via ELISA and Western blot assays. As shown in Figure 3F, the levels of retinol in the mutant retina were decreased by 31%, as compared with WT retina. As illustrated in Figure 3G, mutant retina displayed marked decreases in the levels of STRA6 (retinal vitamin A transport protein) but marked increases in levels in the RPE65 (retinol isomerase), compared with those in the WT retina (Figure 3G) (48–50). These may provide new insights into pathophysiology of LHON-linked mtDNA mutations via the dysregulation of deficient VA metabolism.

### VA supplementation restores retinal deficiency.

**Figure 4:**
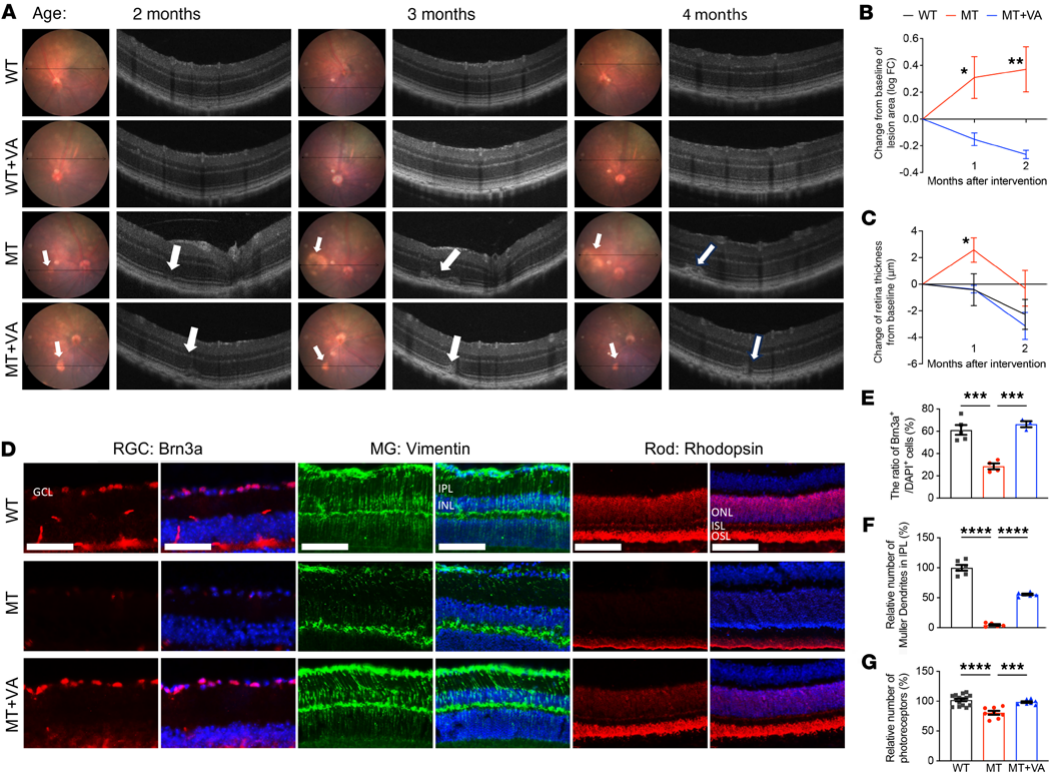
The recovery of retinal deficiency with VA supplementation. (**A**) Image-guided OCT analysis was performed at 2 months (baseline), 3 months, and 4 months of age. The left panel for each age shows the fundus image, and the black arrowed line indicates the location of the OCT scans. The white arrow indicates the fundus lesion. The right panel for each age shows the corresponding OCT images, and the white arrow indicates abnormalities in the photoreceptor and RPE. (**B**) Quantification of changes (logFC) from baseline of lesion areas comparing MT (*n* = 7) and MT+VA mice (*n* = 6). (**C**) Quantification of retinal thickness changes from baseline in WT (*n* = 8), MT (*n* = 6), MT+VA (*n* = 5) mice. (**D**) Immunofluorescence staining of cryosection showing retinal section stained with Brn3a (red) with DAPI (blue) for RGC, Vimentin (green) with DAPI for MG, rhodopsin (red) with DAPI for rod. Scale bar: 50 μm (Brn3a), 100 μm (Vimentin and rhodopsin). GCL, ganglion cell layer; IPL, inner plexiform layer; INL, inner nuclear layer; MG, Müller glia. (**E**–**G**) Quantification of ratios of Brn3a-positive RGC (**E**), relative numbers of Müller dendrites in IPL (**F**), and relative number of photoreceptors (counts of DAPI-positive nuclei in ONL) (**G**) in WT, MT, and MT+VA mouse retina. *n* = 3–5 for Brn3a staining; *n* = 5–6 for Vimentin staining; *n* = 8–15 for photoreceptor counts. Data in **B** and **C** are shown as mean ± SEM. *P < 0.05, **P < 0.01 by 2-tailed unpaired Welch’s *t* test comparing MT and MT+VA mice. Data in **E**–**G** are shown as mean ± SEM. ***P < 0.001, ****P < 0.0001 by 1-way ANOVA followed by Tukey post hoc test.

VA supplementation may be utilized as one therapeutic approach to restore retinal deficiency arising from LHON-linked mtDNA mutation. We investigated if that VA supplementation improved deficient VA metabolism, rescued mitochondrial morphology and function, and subsequently restored retinal deficiencies due to LHON-linked ND6 mutation in the mutant and WT mice. Starting from the age of 2 months, WT and ND6^P25L^ mice were maintained on either a standard VA control chow (4 IU retinol/g) or VA excess chow (120 IU retinol/g). We then evaluated retinal phenotypes at the age of 2 months (baseline) and subsequently at 3 and 4 months. At the age of 2 months, fundus examinations revealed ocular lesions in the mutant retina, a typical phenotype of RPE abnormalities (Figure 4A and Supplemental Table 3) (5, 51). Corresponding optical coherence tomography (OCT) data verified structural abnormalities in the RPE and photoreceptor cells. Notably, the retinal deficiencies in the mutant mice worsened, such as enlarged areas of lesion and increased retina thickness, at the ages of 3 and 4 months (Figure 4, A–C). After supplementing VA for 1 or 2 months, retinal deficiency of ND6^P25L^ mice was significantly restored, including decreased lesion area and retina thickness, as compared with those without VA. In contrast, there were no significant differences of retinal phenotype in the WT mice with and without VA supplementation.

We further investigated the cell-specific effects of VA supplementation on RGCs, Müller cells, and rods. Immunofluorescence staining analysis revealed a 53.4% decrease in Brn3a-positive (transcription factor essential for RGC survival) RGCs, a 95.4% reduction in Vimentin-positive Müller cell neurites in the inner plexiform layer, and a 19.4% decrease in the photoreceptor cells in the mutant mice at the age of 4 months, as compared with WT mice (Figure 4, D–G). Two months of VA supplementation markedly restored the degeneration of retinal cells in mutant mice to levels comparable to those in WT mice (Figure 4, D–G). In particular, the numbers of Brn3a-positive RGCs, Vimentin-positive Müller cell neurites, and the photoreceptor cells in the mutant mice after supplementation of VA were elevated to 233%, 1,204%, and 122% of those in untreated mutant mice. Furthermore, untreated mutant mice exhibited concentrated rhodopsin at the outer segment layer adjacent to the RPE, with a substantial reduction in rhodopsin within the outer nuclear layer (ONL) and inner segment layer (ISL). In contrast, rhodopsin distribution in VA-supplemented ND6^P25L^ mice was similar to that in WT mice (Figure 4D). These results indicated that dietary VA supplementation remarkably ameliorated retinal abnormalities and cellular deficiencies in ND6 mutant mice.

### Restoration of VA metabolism and visual function.

**Figure 5:**
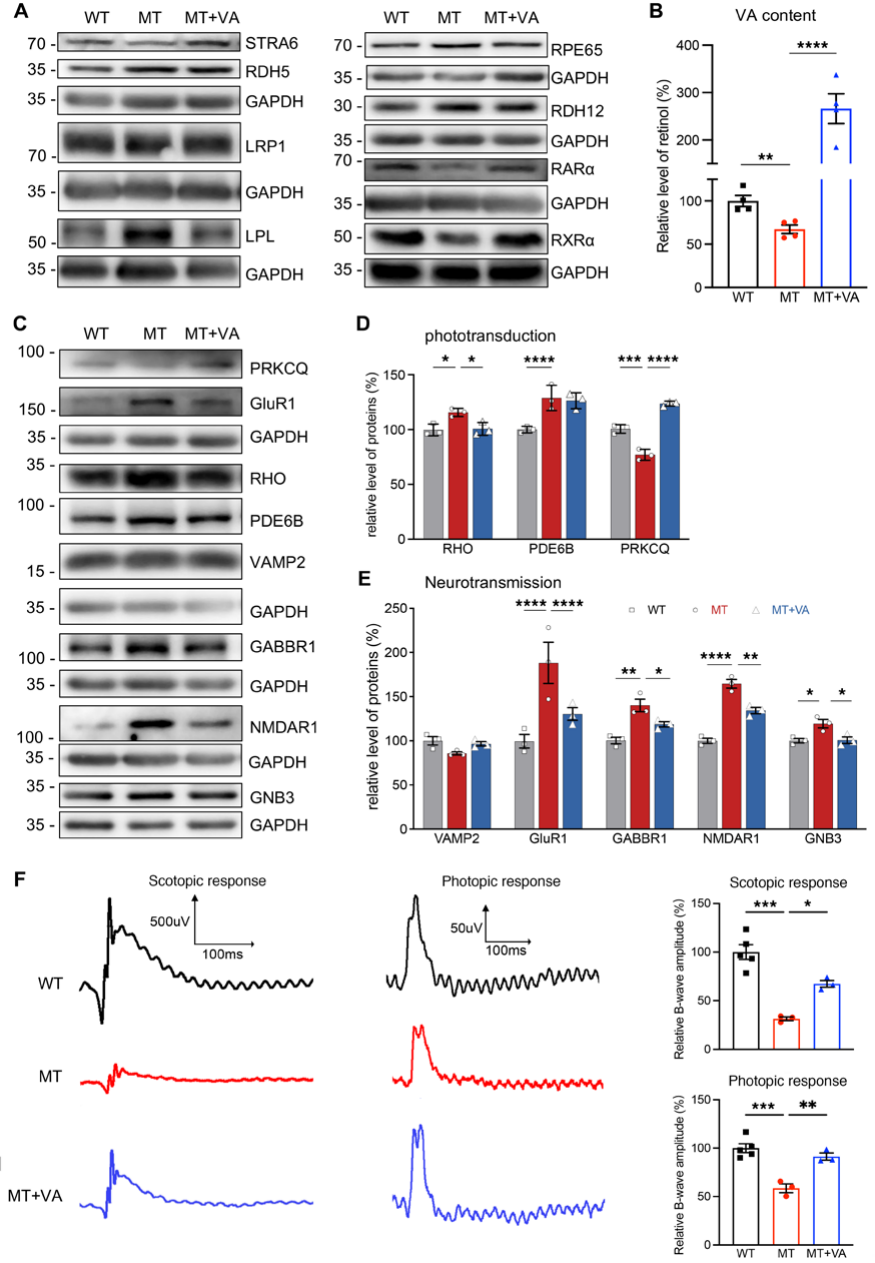
VA metabolism and visual function. (**A**) Western blot analysis of proteins involved in VA metabolism. Total cellular proteins in WT, MT, and MT+VA retina were electrophoresed with PAGE and hybridized with STRA6, RDH5, LRP1, LPL, RPE65, RDH12, RARα, and RXRα antibodies and GAPDH as a loading control, respectively. (**B**) Relative levels of retinol in eyeballs of WT, MT, and MT+VA mice using ELISA. *n* = 4 mice per group; **P < 0.01, ****P < 0.0001 by 2-tailed unpaired Student’s *t* test of the differences between MT and WT, or MT and MT+VA mice. Normality was assessed using the Shapiro-Wilk test (*P* > 0.05), and equal variance was confirmed using the *F* test (*P* > 0.05). (**C**) Western blot analysis of proteins involved in phototransduction and neurotransmission. Total cellular proteins in WT, MT, and MT+VA retina were electrophoresed with PAGE and hybridized with RHO, PRKCQ, GluR1, PDE6B, VAMP2, GABBR1, NMDAR1, and GNB3 antibodies and GAPDH as a loading control, respectively. (**D** and **E**) Quantification of RHO, PDE6B, and PRKCQ for phototransduction (**D**) and VAMP2, GluR1, GABBR1, NMDAR1, and GNB3 for neurotransmission (**E**) in WT, MT, and MT+VA retina. The calculations were based on 3 independent determinations in each mouse. (**F**) Analysis of ffERG for WT (*n* = 5), MT (*n* = 3), or MT+VA (*n* = 3) mice. By dark adaptation for a night, mice were analyzed for scotopic response and then photopic response. Data in **D**–**F** are shown as mean ± SEM. *P < 0.05, **P < 0.01, ***P < 0.001, ****P < 0.0001 by 1-way ANOVA followed by Tukey’s post hoc test.

We assessed if the VA supplementation restored the deficient VA metabolism due to ND6 mutation. The levels of VA metabolic pathway–related proteins, including STRA6, RPE65, retinol dehydrogenases (RDH5 and RDH12), lipoprotein ligase (LPL), and lipoprotein receptor (LRP1), in the presence and absence of VA supplementation were measured by Western blot analysis (28–30). As shown in Figure 5A and Supplemental Figure 5A, the VA supplementation levels made the levels of STRA6, RPE65, and LPL in the mutant retina comparable to those in WT mice but did not change the levels of RDH5, RDH12, and LRP1 in the mutant retina. Furthermore, the VA treatment elevated the levels of retinoid acid (RA) receptors RARα and RXRα in the mutant retina, indicating the recovery of VA signaling pathways (Figure 5A and Supplemental Figure 5A) (52). As shown in Figure 5B, ELISA revealed that VA supplementation increased VA content in the eyes of ND6^P25L^ mice to 395.3% of the levels without VA supplementation.

We then investigated whether VA supplementation ameliorated the phototransduction and neurotransmitter transmission pathways in the mutant mice. The administration of VA resulted in various changes in the levels of components (RHO, PRKCQ, and PDE6B) of phototransduction pathways and components (GluR1, GABBR1, NMDAR1, GNB3, and VAMP2) of neurotransmitter transmission pathways (Figure 5, C–E, and Supplemental Figure 5B) (2, 53–55). In particular, VA administration resulted in decreased RHO levels and increased PRKCQ levels but no change in PDE6B level in the mutant retina (Figure 5, C and D). Moreover, markedly increased levels of GluR1, GABBR1, NMDAR1, and GNB3 occurred in the mutant retina, but VA administration reduced the levels of these proteins to be comparable to those in WT retina (Figure 5, C and E). However, VA treatment did not significantly change the levels of VAMP2, associated with the glutamate neurotransmitter release pathway in the mutant retina, as compared with those in WT retina (Figure 5, C and E).

We then evaluated if VA supplementation restored visual functions, focusing on photoreceptor deficits, by full-field electroretinography (ffERG). As shown in Figure 5F, the amplitudes of b-waves for scotopic (rod) responses and photopic (cone) responses in the mutant retina were 31.5% and 58.6% of those in WT mice. The supplementation of VA increased these amplitudes to 213.9% and 155.6% of unsupplemented levels for scotopic responses and photopic responses, respectively. These data demonstrated that the VA supplementation restored retinal failure arising from ND6 mutation.

### Rescuing abnormal mitochondrial morphology and function.

**Figure 6:**
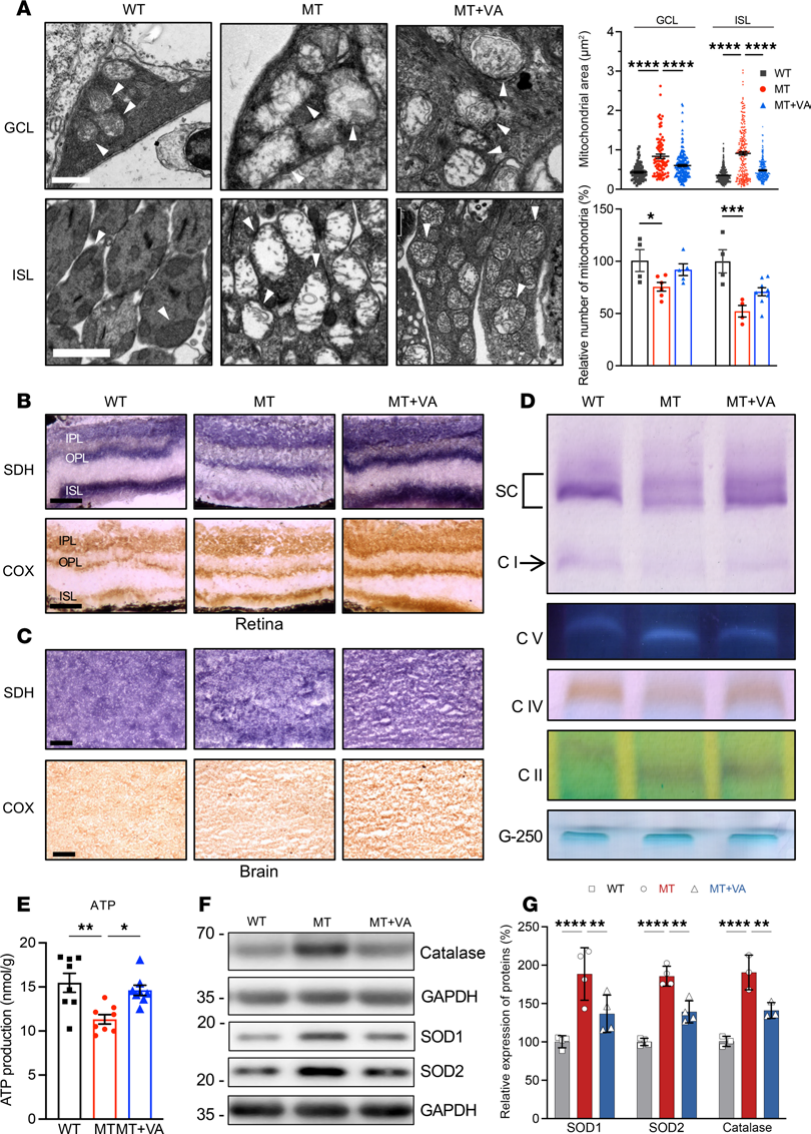
Mitochondrial morphology and function. (**A**) Representative transmission electron micrographs of mitochondria from GCL and ISL of retina in WT, MT, and MT+VA mice. Scale bars: 1 μm in GCL, 2 μm in ISL. The arrow indicates mitochondria. The right panel shows the quantification of mitochondrial size (*n* = 86–319 mitochondria) and relative mitochondrial number (*n* = 4–6 mice) in GCL and ISL of mouse retina. (**B** and **C**) Assessment of mitochondrial function by enzyme histochemistry staining for COX and SDH in the frozen sections of retinas (**B**) and brains (**C**) in WT, MT, and MT+VA mice. scale bar: 50 μm in retina; 100 μm in brain. OPL, outer plexiform layer. (**D**) In-gel activity of respiratory chain complexes I, II, IV, and V. Twenty micrograms of mitochondrial protein from brains of WT, MT, and MT+VA mice was used for BN-PAGE, and the activities of complexes were measured in the presence of specific substrates. Coomassie staining was used as a loading control. SC, super complexes. (**E**) ATP levels among brains of WT (*n* = 8), MT (*n* = 8), and MT+VA (*n* = 8) mice were measured using a luciferin/luciferase assay. Absolute level of total cellular ATP was shown. (**F**) Western blot analysis of antioxidant proteins. Total cellular proteins in WT, MT, and MT+VA retinas were electrophoresed with PAGE and hybridized with catalase, SOD1, and SOD2 antibodies and GAPDH as a loading control, respectively. (**G**) Quantification of catalase, SOD1, and SOD2 in WT, MT, and MT+VA retina. Representative of 3 to 4 independent experiments. Data in **A**, **E**, and **G** are represented as mean ± SEM. *P < 0.05, **P < 0.01, ***P < 0.001, ****P < 0.0001 by 1-way ANOVA followed by Tukey’s post hoc test.

We then assessed if VA supplementation was able to rescue ND6 mutation–induced abnormal morphology and function of mitochondria in the mouse retina. As shown in Figure 6A, supplementation of VA significantly corrected abnormal mitochondrial morphologies, including swelling, cristae malformations, enlarged size, and reduced number in the RGCs and rods of mutant retina, as compared with WT retina.

We then examined if the VA administration restored mitochondrial dysfunctions in the retina by enzyme histochemistry staining for succinate dehydrogenase (SDH) and cytochrome *c* oxidase (COX) in the frozen retinal sections. As shown in Figure 6B, mutant retina exhibited markedly decreased SDH activity, particularly in the ISL, but mild reductions in COX activity, as compared with those in WT retina. In contrast, the SDH and COX staining in the brains between mutant and WT mice was not significantly different (Figure 6C). The discrepancy of complex II between the brain and retina was further assessed by Western blot analysis of complex I and II subunits. As shown in Supplemental Figure 6A, the levels of SDHA and SDHB were decreased in retina but not changed in the brain in the mutant mice, as compared with those in WT mice. We then analyzed the activities of OXPHOS complexes I, II, IV, and V using in-gel activity assays. Due to the difficulty in obtaining enough mutant mouse retinas, we used brains, which belong to the central nervous system and may share some common mitochondrial features with retina (56), for these experiments. Mitochondrial membrane proteins isolated from brains were separated by blue native–PAGE (BN-PAGE) and stained with specific substrates for each complex. As shown in Figure 6D, the brains of mutant mice exhibited markedly decreased activities of complexes I and IV but no changes in those of complexes II and V, as compared with WT mice. Furthermore, the levels of total cellular ATP production in the mutant brains were 73% of those in the WT brains (Figure 6E). Strikingly, the supplementation of VA remarkably elevated SDH and COX activities in the mutant retina (Figure 6B) and increased activities of complexes I and IV as well as levels of total cellular ATP in the mutant brains (Figure 6, D and E).

To test whether VA supplementation reduced the overproduction of ROS in the mutant retina, we measured the levels of antioxidant-related proteins, including catalase, SOD1, and SOD2, in mouse retina (45). As shown in Figure 6, F and G, the levels of SOD1, SOD2, and catalase in the mutant retina were 188.6%, 186.7%, and 190.5% of those in WT retina. Notably, VA supplementation gave rise to significantly reduced levels of SOD1, SOD2, and catalase in the mutant retina, indicating that VA supplementation reduced overproduction of ROS. The levels of ROS production were further measured by dihydroethidium staining in the frozen brain sections. As shown in Supplemental Figure 6B, increased levels of dihydroethidium staining reflecting the levels of ROS were observed in the mutant brains, and VA supplementation reduced the levels of dihydroethidium staining in the mutant brains, as compared with WT brains.

## Discussion

In this study, we investigated the retinal cell–specific effects and therapeutic potential of VA supplementation in LHON using a mouse model bearing the LHON-linked ND6^P25L^ mutation. This mouse model bearing the ND6^P25L^ mutation revealed the degeneration of RGCs and retinal nerve fiber loss, similar to retinal changes seen in human patients with LHON (13, 14, 27). However, this rodent model also featured significant degenerations of other retinal cells including rods and Müller cells, which are not described as prominent features of human patients with LHON (13, 14). These retinal cell–specific defects in the mutant mice were further verified by the scRNA-Seq data that the mutant retina exhibited various reductions in neuronal cell types, including cones, bipolar cells, horizontal cells, and Muller2 cells, and especially pronounced effects on Rod2 cells. Furthermore, functional defects in rods were corroborated by significant declines in dark-adapted amplitudes of b-waves for scotopic responses of ND6^P25L^ mouse eyes (Figure 5F). Indeed, characterization of these phenotypes in human patients has been challenging because of the difficulty in accessing patients’ tissues. Despite the organizational and metabolic differences between mouse and human retina, these findings may impact the precision diagnosis and treatment of LHON.

The ND6^P25L^ mutation led to the retinal cell–specific effects of OXPHOS biogenesis and subsequent failure of cellular energetic process. scRNA-Seq analysis revealed the dysregulation of OXPHOS pathways among the majority of cell clusters in mutant retina, including upregulation of mitochondrial tRNA processing and TCA cycle and downregulation of electron transport chains and ATP synthase, similar to biochemical phenotypes in cellular and mouse disease models (25, 57–60). As a result, these defects resulted in the deficient assembly and activity of OXPHOS complexes and increased overproduction of ROS in the mutant retina. Strikingly, ND6 mutation caused the cell-specific transcriptional dysregulation in the genes involved in OXPHOS biogenesis within the retina. In particular, much more downregulation than upregulation of these genes occurred in RGCs, while predominantly upregulation of these genes was observed in cone, HC, GABAergic AC, Rod2, and Muller2. This discrepancy may be attributed to retinal cell–specific energy demands and potential compensatory responses (61–64). These cell-specific mitochondrial dysfunctions were validated by various reductions in the SDH staining in the mutant retina, particularly pronounced decreases in rods, in contrast with no difference in the SDH staining between mutant and WT brains. However, the ND6 mutation downregulated the expression of other mtDNA-encoded complex I subunits in both retina and brain, which belong to the central nervous system and share some common mitochondrial features (56). Thus, the reduced activity of complex I in the brain mirrors the complex I deficiency in the retina in the mutant mice. The discrepancy of complex I and complex II activities between the brain and retina may reflect the tissue-specific mitochondrial function and metabolism. Furthermore, the differing elevating expression of antioxidant genes and ROS detoxification enzymes in most cell types, especially in the RGCs, reflected the cell-specific oxidative stress induced by mitochondrial dysfunctions (65–68).

The exciting discovery in the present investigation is that ND6^P25L^ mutation caused abnormalities in VA metabolism essential for vision. VA metabolism is involved in VA transfer to and storage in RPE, transfer to the photoreceptors, conversion to its active form, recycling of the inactive forms, and removal of the toxic by-products (28, 30, 69). In fact, VA also plays a role in the mitochondrial biogenesis by regulating the expression of both mtDNA and nucleus-encoded OXPHOS subunit (31–33). Hence, it is very likely that VA metabolism has feedback from deficient OXPHOS biogenesis arising from ND6 mutation and defective nuclear genes, such as *PRICKLE3* and *NDUFS4* (25, 59). In this study, scRNA-Seq analysis revealed significant upregulation of the VA metabolic pathway (Figure 3C), specifically in converting VA to active form and recycling of the inactive forms, in the mutant retina. In particular, the downregulation of STRA6 involved in transporting VA from blood into RPE and upregulation of RPE65 converting all-*trans*-retinol into 11-*cis*-retinol in the mutant retina reflected defective VA transfer to RPE and conversion to its active form, as in the case of *STRA6*-knockout mice (70–72). Furthermore, the upregulation of LPL, which hydrolyzes triglycerides and retinyl esters within chylomicrons and is one component of other pathways for VA transport, occurred in mutant retina, suggesting an adaptation of the retinal environment to VA deficiency in mutant mice (69). Moreover, the levels of RARα and RXRα, downstream markers of VA signaling pathways, were drastically decreased in the mutant retina. The dysregulation of RA signaling pathways may result in the low expression of STRA6, an RA-responsive gene (49, 52), and then exacerbate VA deficiency in the retina. The deficient VA metabolism led to reductions in VA content in the mutant eye and alterations in photoreceptor and RPE in the mutant eye (51, 72, 73). Other retinopathies arising from mitochondrial dysfunctions, such as progressive external ophthalmoplegia, macular pattern dystrophy, pigmentary retinopathy, and optic neuropathy, may similarly be driven by VA transport failure in the RPE. These findings indicated the role of VA and its derivatives in the pathophysiology of LHON and other mitochondrial retinopathies, and their potential as therapeutics.

The development of effective therapeutic strategies for treatment or cure of LHON has been challenging due to the complex genetics, manifested by mtDNA mutation and mutated nuclear modifiers or epigenetic factors (4, 74, 75). In this study, we demonstrated that VA administration restored mitochondrial dysfunctions and vision failure in the mutant retina. This treatment alleviated retinal deficiencies, including reductions in fundus lesion and retinal thickness and increases in the numbers of RGCs, photoreceptors, and Müller cell neurites. The restoration of visual functions was further supported by correcting dysregulations of phototransduction cascade and neurotransmitter transmission and restoring abnormal electrophysiological properties. In fact, the VA and its derivatives mediate mitochondrial dynamics and exert effects on energy metabolism and cell function (31, 33, 76–78). Here, the VA administration corrected abnormal mitochondrial morphology, including reduced sizes and increased numbers of mitochondria in RGCs and rod cells in the mutant mice, indicating the role of VA in mitochondrial dynamics (76). Furthermore, VA administration exerted effects on OXPHOS biogenesis and restored mitochondrial functions, evidenced by increasing expression of OXPHOS subunit, activity of complex I, and ATP production and reducing overproduction of ROS in the mutant mice. However, the VA dose used in the mouse model was more than 30 times higher than the control dose, exceeded the maximum safe daily intake dose for humans, and potentially caused acute and chronic side effects. The differences in VA transport and metabolism may result in differential effects of dietary VA supplementation between humans and rodents (79, 80). Further studies are necessary to identify the optimal dose of VA to treat patients with LHON. Our finding may open a new avenue to therapeutic interventions for LHON and other mitochondrial retinopathy, including RPE-related disorders such as macular degeneration, retinitis pigmentosa, and Stargardt’s disease (5).

In summary, our investigation demonstrated that the ND6^P25L^ mutation led to cell-specific defects in the retina, including marked degeneration in RGCs, and deficiencies in other retinal cells, including rods and Müller cells. The ND6^P25L^ mutation caused retinal cell–specific transcriptional regulation of OXPHOS pathways, dysregulation of visual signaling pathways, abnormal VA metabolism, and consequently visual failure. The supplementation of VA yielded the morphological and functional recovery of mitochondria and retinal cells in the mutant mice. These findings provide insights into the pathophysiology of LHON arising from mtDNA mutation–induced retinal cell–specific deficiencies and abnormal VA metabolism and a step toward therapeutic intervention for LHON and other mitochondrial retinopathies.

### Strengths.

The mouse disease model is an excellent disease model for deeply elucidating the pathophysiology of this disease. The mouse model bearing the ND6^P25L^ mutation exhibited retinal cell–specific manifestations similar to retinal changes seen in human patients with LHON, such as RGC deficiency, and never described in human patients with LHON, such as degenerations of other retinal cells, including rods and Müller cells. The LHON-linked mtDNA mutation caused the deficient VA metabolism, and the administration of VA rescued the retinal deficiency arising from LHON-associated mtDNA mutation.

### Limitations.

The mouse model carrying the homoplasmic ND6^P25L^ mutation across whole body manifested not only retinal cell–specific deficiencies but also in other tissues, such as cardiac defects. The mechanism underlying the mitochondrial dysfunction–induced deficient VA metabolism needs to be further investigated. This study lacks the clinical trial data, especially the appropriate dose of VA, to treat patients with LHON.

## Methods

### Sex as a biological variable.

Our study examined male mice because LHON is clinically more prevalent in males. Additionally, due to the reproductive challenges of mtDNA-mutant mice, female mice were primarily used for breeding purposes.

### Mouse genetics.

All animal care protocols were approved by the Animal Care and Use Committee of Zhejiang University School of Medicine. C57BL/6J WT mice were originally purchased from Shanghai SLAC Laboratory Animal Co, Ltd. Sanger sequencing analysis of the *Crb1* gene failed to detect the Rd8 mutation in vendor lines of C57BL/6J mice (81). The mice harboring the mtDNA ND6 13997G>A P25L mutations were provided by the University of Pennsylvania, and the mice were generated via the female embryonic stem cell fusion method (27, 82). The WT mice were maintained by brother-sister matings. The mtDNA ND6^P25L^ mutant mice were maintained by crossing female mtDNA-mutant mice with WT males. For genotyping ND6^P25L^ mice, a PCR segment (825 bp) spanning the ND6 gene was amplified from mouse tail DNA using primers (5′-CCCCCTAAATAAATTAAAAAA-3′ and 5′-ATAATAAATGGTAAGATGAAG-3′) and analyzed by Sanger sequencing. The resulting sequence data were compared with the updated consensus sequence (GenBank accession number: NC_005089.1) (83). All mice used for experiments were matched for sex and age.

### Library preparation for scRNA-Seq.

Libraries for scRNA-Seq were generated using the Chromium Single Cell 3′ library and Gel Bead & Multiplex Kit from 10x Genomics. We aimed to profile 8,000 cells per library (if sufficient cells were retained during dissociation). All libraries were sequenced on Illumina NovaSeq 6000. After quality control, raw sequencing reads were aligned to the mouse reference genome mm10 and processed to a matrix representing the unique molecular identifiers (UMIs) per cell barcode per gene using Cell Ranger (v3.0.2, 10x Genomics).

### Single-cell gene expression analysis.

Raw gene expression matrices generated per sample were merged and analyzed with the Seurat package (v3.1.4). Cell matrices were filtered by removing cell barcodes with <401 UMIs, <201 expressed genes, or >20% of reads mapping to mitochondrial RNA. The remaining cells were normalized and the 3,000 most variable genes were selected to perform a principal component analysis (PCA) after regression for confounding factors: number of UMIs, percentage of mitochondrial RNA, patient ID, cell cycle (S and G2M phase scores calculated by the CellCycleScoring function in Seurat), interferon response (BROWNE_INTERFERON_RESPONSIVE_GENES in the Molecular Signatures Database v6.2), and sample dissociation-induced stress signatures. To reduce batch effect, we performed data integration using anchor-based canonical correlation analysis in Seurat (v3) package between samples, followed by PCA and graph-based clustering. Clusters were calculated by the FindClusters function with a resolution between 0.2 and 2 and visualized using the t-distributed stochastic neighbor embedding dimensional reduction method. Differential gene expression analysis was performed for clusters generated at various resolutions by both the Wilcoxon rank sum test and Model-based Analysis of Single-cell Transcriptomics using the FindMarkers function. A specific resolution was selected when known cell types were identified as a cluster at a given resolution, but not at a lower resolution, with the minimal constraint that each cluster had at least 10 significantly differentially expressed genes (FDR < 0.01 with both methods) with at least a 2-fold difference in expression compared with all other clusters. Annotation of the resulting clusters to cell types was based on the expression of marker genes.

### SCENIC analysis.

Transcription factor (TF) activity was analyzed using SCENIC (v1.0.0.3) per cell type with raw count matrices as input. The regulons and TF activity (AUC) for each cell were calculated with the pySCENIC (v0.8.9) pipeline with motif collection version mc9nr. The differentially activated TFs of each subcluster were identified by the Wilcoxon rank sum test against all the other cells of the same cell type. TFs with logFC > 0.1 and an adjusted *P* < 1 × 10^−5^ were considered as significantly upregulated.

### Modeling of metabolic pathways based on scRNA-Seq data.

The metabolic landscape of mouse retina was modeled using the Compass method (version 0.9.5) (47) by leaving the standard settings unaltered. The gene expression matrix of retinal single-cell data was used as input. The Compass output data were concatenated and transformed as described (47). To determine which reactions and metabolites were significantly different between groups (WT and MT), a Wilcoxon rank sum test on Compass scores was performed.

### Quantitative real-time reverse transcription PCR.

Total RNA isolation and qRT-PCR were carried out as described previously (84). Briefly, the first-strand cDNA synthesis was carried out using a reverse transcription system kit according to the instructions of the manufacturer (Promega). qRT-PCR was performed in a 7900 HT Fast Real-time PCR system (Applied Biosystems). The mRNA levels of various genes were calculated after normalizing with *β-actin*. Primer sequences used for this analysis are presented in the Supplemental Table 4.

### Western blotting.

Western blot analysis was performed using 20 μg of total cellular proteins isolated from mouse tissues, as described elsewhere (21, 85). Samples were run through a 10% bis-Tris SDS-PAGE and electroblotted onto a PVDF membrane. The antibodies used for this investigation are summarized in Supplemental Table 5. Peroxidase AffiniPure goat anti-mouse IgG and goat anti-rabbit IgG (Beyotime A0216 and A0208, respectively) were used as secondary antibodies, and protein signals were detected using the ECL system (CWBIO). The quantification of density in each band was performed, as detailed previously (21, 85).

### VA measurement.

Eyes were enucleated and 2 eyecups from individual mice were pooled and homogenized in 200 μL hydroxylamine. VA concentration of eyecups was detected by an ELISA-based kit (Elabscience Biotechnology Co. Ltd.).

### VA supplementation.

VA excess chow and VA control chow were purchased from Xietong Shengwu. The only difference between these two types of chow is the concentration of VA, with 120,000 IU/kg in the VA excess chow and 4,000 IU/kg in VA control chow. The dose of VA with 120,000 IU/kg used for this investigation was in reference to the previous observation that the long-term administration of this megadose of VA rescued vision failure in a preclinical mouse model for RhoD190N-associated retinitis pigmentosa (86, 87). The WT and ND6-mutant mice were divided into 4 groups: wild-type mice on control chow (WT), wild-type mice on VA excess chow (WT+VA), ND6^P25L^ mice on control chow (MT), and ND6^P25L^ mice on VA excess chow (MT+VA). WT mice and ND6^P25L^ mutant mice at the age of 2 months were randomly assigned to one diet. All mice were maintained on either diet with ad libitum access to food and water on a 12-hour light/12-hour dark cycle for 8 weeks for longitudinal analysis of fundus phenotype.

### Fundus imaging and OCT.

An SD-OCT system (Micron IV, Phoenix Research Laboratories) was applied for fundus imaging and thickness measurements of the retinal layers of 2-month-old mice as baseline and months 1 and 2 after dietary intervention. Mice were anesthetized with pentobarbital sodium, and pupils were dilated with 1% tropicamide (Bausch & Lomb) followed by application of GenTeal Lubricant Eye Gel (Alcon). Systane lubricant eye drops (Alcon) were applied to keep the cornea moist. The fundus was viewed with an imaging camera, and SD-OCT with guidance of bright-field live fundus image was performed using the image-guided OCT system according to the manufacturer’s instruction. The retinal layer thickness was analyzed semiautomatically with Inviewer 3.0.

### Immunofluorescence.

Eye cups were dissected and immersed in 4% paraformaldehyde in PBS for 24 hours, then embedded in O.C.T. compound (Thermo Fisher Scientific) after infiltrating with 30% sucrose overnight. Transverse sections of the retina (10 μm thick) were mounted onto slides and blocked with 20% fetal calf serum in PBS for 1 hour. The retinas were incubated with primary antibodies at 4°C overnight, then the secondary antibodies. Images were taken by Leica DM4000B-M and Olympus Fluoview FV1000 microscopes. The antibodies used for this investigation are summarized in Supplemental Table 5.

### ERG recordings.

ERGs of 4-month-old mice were recorded as previously described (88). Both the scotopic and photopic ERGs were recorded with a well-established Ganzfeld Q450 dome stimulating and recording system. Mice were dark-adapted for 12 hours. Under dim red light, mice were anesthetized with intraperitoneal injection of ketamine/xylazine (80 mg/kg, 10 mg/kg, respectively). Pupils were dilated with tropicamide and corneas were protected by application of a thin layer of methylcellulose. ERGs were recorded via coiled silver electrodes contacting the moist cornea. A gold needle electrode was placed under the skin between shoulders to serve as both reference and ground. Responses were amplified differentially, band-pass filtered (0.1 to 500 Hz), and digitized at 10 kHz. Responses to flashes were averaged with an interstimulus interval ranging from 2 seconds for dim lights to 10 seconds for the brightest flashes. Five responses were averaged for each light intensity to eliminate electrical noise. Single-flash cone response was recorded after 10 minutes of light adaptation. ERGs were performed using a 0.01 and 1 cd·s/m^2^ flash stimulus and a 10 cd·s/m^2^ and 20 Hz flicker stimulus, respectively.

### Retinal ultrastructure and mitochondrial morphology.

The ultrastructure of mouse retina at 2 months after intervention was observed using transmission electron microscopy. Retina tissues were enucleated and fixed in 2.5% glutaraldehyde overnight. The specimens were postfixed with 1% OsO_4_ for 1 hour at room temperature, stained and blocked with 2% uranyl acetate for 30 minutes, and embedded in Epon 812 after dehydration. The sample was cut into 100 nm thick slices using UC7 ultramicrotome (Leica), and finally the images of retina ultrastructure were captured by a TECNAI transmission electron microscope (Philips).

### Native gel electrophoresis and enzymatic activity assays.

The activity of mitochondrial respiratory complexes was analyzed by mitochondria isolated from mouse brains using BN gel electrophoresis as detailed elsewhere (59). In brief, 400 μg mitochondria were solubilized in 0.5% DDM solution containing 50 mM NaCl, 50 mM imidazole, 2 mM 6-aminohexanoic acid, and 1 mM EDTA (pH 7.4) on ice for 20 minutes. After removing insoluble material by centrifugation at 12,000*g* for 20 minutes, Coomassie Brilliant Blue G-250 (Serva) was added to 20 μg lysed mitochondria at a dye/detergent ratio of 1:5 (w/w). The sample was loaded onto 3%–11% acrylamide gradient gels and electrophoresed at 150 V in dark-blue cathode buffer for 1 hour and then 250 V in light-blue running buffer for 1.5 hours at 4°C. The native gels were prewashed in cold water and then incubated with the substrates of complex I, complex II, complex IV, and complex V at room temperature: NADH and NTB for complex I; sodium succinate, phenazine methosulfate, and NTB for complex II; DAB and cytochrome *c* for complex IV; and glycine, MgSO_4_, ATP, and Pb(NO3)_2_ for complex V, as described elsewhere (84).

### COX-SDH histochemistry.

Enzyme histochemistry staining for SDH and COX in the frozen sections from mouse retinas was performed as detailed elsewhere (25). Briefly, freshly dissected retina tissues were embedded in OCT compound (Tissue-Tek), frozen on dry ice, and sectioned to 8 μm. For SDH staining, samples were incubated in 0.1 M phosphate buffer at pH 7.6, containing 5 mM EDTA, 1 mM potassium cyanide, 0.2 mM phenazine methosulfate, 50 mM succinic acid, and 1.5 mM nitro blue tetrazolium, at 37°C for 25 minutes. For COX staining, samples were incubated in 5 mM phosphate buffer at pH 7.6, containing 5 mM EDTA, 1 mM potassium cyanide, 0.2 mM phenazine methosulfate, 50 mM succinic acid, and 1.5 mM nitro blue tetrazolium, at 37°C for 60 minutes.

### ATP measurement.

The Enhanced ATP Assay Kit (Beyotime) was used for the measurement of total cellular ATP levels in mouse brains, according to the manufacturer’s instructions with some modification, as described previously (25).

### ROS measurement.

ROS levels were assessed using dihydroethidium (DHE; S0063, Beyotime, China). Fresh brain tissues were embedded in OCT compound (Tissue-Tek) and frozen on dry ice. Transverse brain sections (10 μm thick) were mounted onto slides and incubated with 5 μM DHE solution at room temperature for 30 minutes. Following incubation, the sections were rinsed 3 times with PBS. ROS fluorescence images were captured using a fluorescence microscope (Leica DM4000B-M). DHE red fluorescence was detected with an excitation wavelength of 525 nm and an emission wavelength of 590 nm, using identical exposure settings across samples. The intensity of red fluorescence was used as a measure of ROS levels, with higher fluorescence indicating greater ROS content.

### Statistics.

All data were expressed as mean ± SEM if not specified otherwise in the legends. Statistical analysis of the data was performed using GraphPad Prism 9. Comparison between 2 groups was made using a 2-tailed unpaired Student’s *t* test. Normality was assessed using the Shapiro-Wilk test (*P* > 0.05), and equal variance was verified using the *F* test (*P* > 0.05). In cases where the *F* test indicated unequal variances, Welch’s *t* test was used as an alternative to the Student’s *t* test. The statistical significance for multiple groups was assessed by 1-way ANOVA, followed by the Tukey post hoc test. Results are presented in dot plots, with dots denoting individual values. *P* < 0.05 was considered statistically significant. Further statistical details of experiments can be found in the figure legends and Results section.

### Study approval.

All animal experiments were approved by the Institutional Animal Care and Use Committee, Zhejiang University School of Medicine.

### Data availability.

Raw scRNA-Seq data are available at the National Center for Biotechnology Information Gene Expression Omnibus with the accession number GSE280785. A Supporting Data Values file is provided as supplemental material.

## Author contributions

MXG designed the experiments and monitored the project progression and data analysis and interpretation. CA and HL performed the mouse experiments; JQ, CA, and CW carried out scRNA-Seq data analysis; DCW provided the mouse model; CA, YJ, and JQ undertook the data analysis; YZ acquired funds; CA and JQ prepared the initial draft of the manuscript; and MXG prepared the final version of the manuscript. All authors reviewed the manuscript.

## Supplementary Material

Supplemental data

Unedited blot and gel images

Supporting data values

## Version 1 03/04/2025

In-Press Preview

## Version 2 04/22/2025

Electronic publication
