# Supplementary material for: Vitamin A treatment restores vision failures arising from Leber’s hereditary optic neuropathy–linked mtDNA mutation
Source: JCI Insight. 2025 Mar 4;10(8):e188962. doi: 10.1172/jci.insight.188962 (PMC12038914; doi:10.1172/jci.insight.188962)
Supplement: Supplemental data [file jciinsight-10-188962-s019.pdf]

Supplementary data for ***188962-INS-RG-RV-2***

Vitamin A treatment restores vision failures arising from Leber's hereditary optic neuropathy-linked mtDNA mutation

Cheng Ai[1,2,3#], Huiying Li[2#] , Chunyan Wang[2,4], Yanchun Ji[2,5], Douglas C. Wallace[6], Junbin Qian[2,4*], Yimin Zhu[4] and Min-Xin Guan[1,2,3,7*]

The supplemental data included the following information:

1. Supplemental Figure 1, 2, 3, 4, 5 and 6

2. Supplemental Table 1, 2, 3, 4 and 5

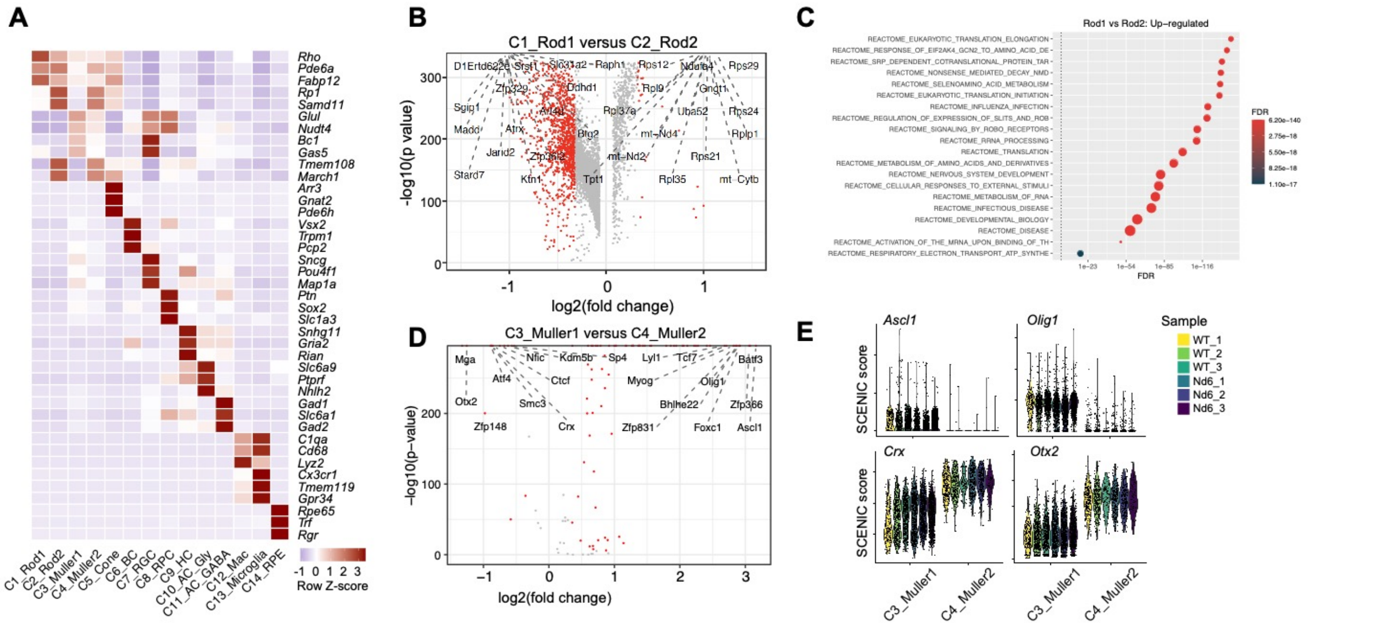

Supplemental Figure 1 (related to Figure 1): Cluster identification in scRNA-seq. (A) Heatmap showing the expression of marker genes for each cell type. **(B)** Volcano plot showing DEGs comparing Rod1 and Rod2. Dots on the volcano plot: gray, no significant change; red, P < 0.05 and absolute log2FC ≥ 0.5. P values were obtained by the model-based analysis of single-cell transcriptomics (MAST) test and Bonferroni-corrected (Seurat). **(C)** Dot plot showing up-regulated REACTOME pathway comparing Rod1 and Rod2. **(D)** Volcano plot showing DEGs comparing Muller1 and Muller2. Dots on the volcano plot: gray, no significant change; red, P < 0.05 and absolute log2FC ≥ 0.5. **(E)** Violin plots of Ascl1, Olig1, Crx, Otx2 expression in Muller1 and Muller2.

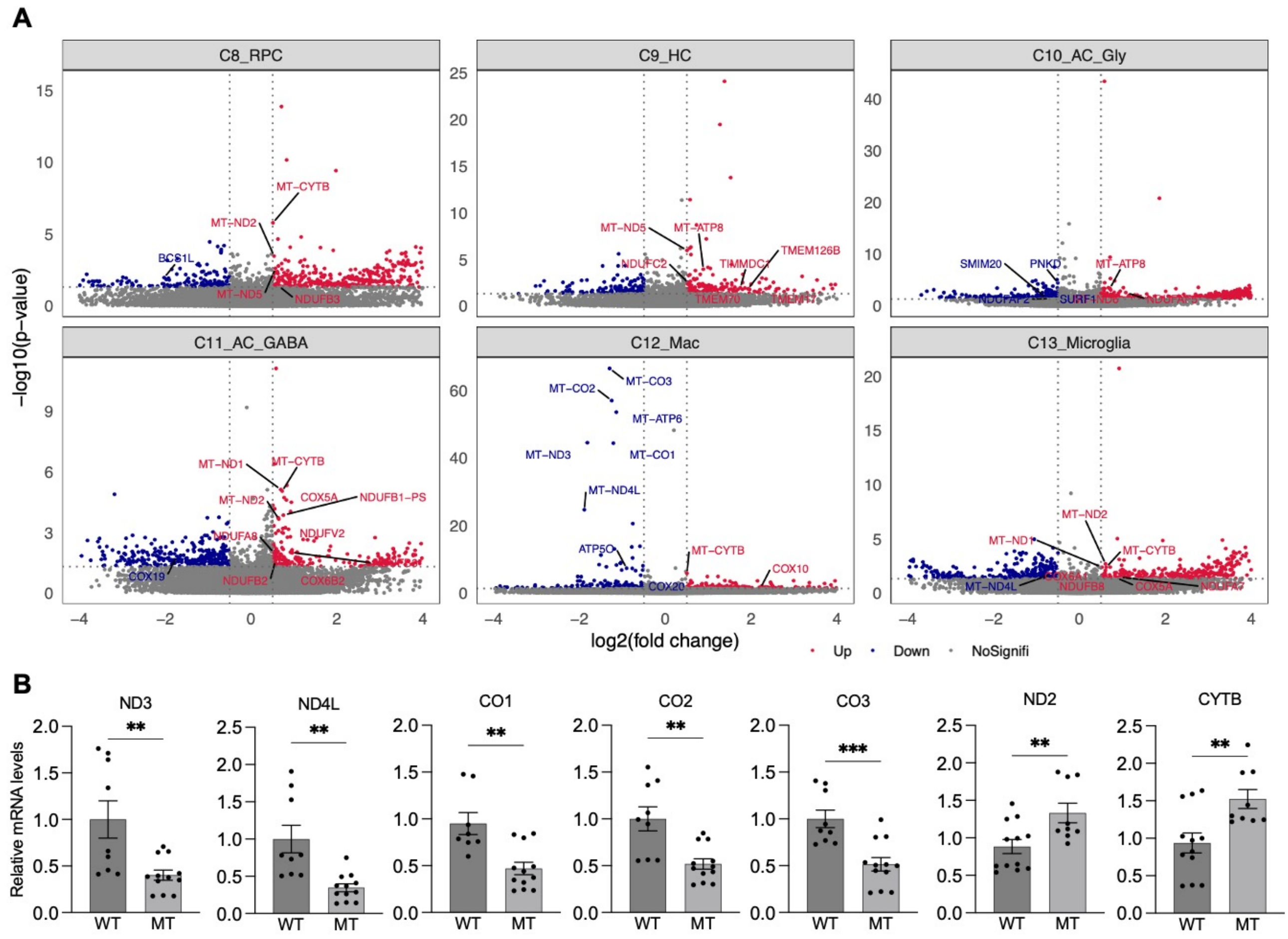

Supplemental Figure 2 (related to Figure 2): Cell-specific OXPHOS gene expression in retina cell clusters. (A) Volcano plot showing differentially expressed genes comparing WT and MT in different clusters (C8_RPC, C9_HC, C10_AC_Gly, C11_AC_GABA, C12_Mac and C13_Microglia). Dots on the volcano plot: gray, no significant change; red, $P < 0.05$ and log2FC ≥ 0.5; blue, $P < 0.05$ and log2FC ≤ -0.5. The significantly differentially expressed OXPHOS genes were labeled. *P* values were obtained by the model-based analysis of single-cell transcriptomics (MAST) test and Bonferroni-corrected (Seurat). **(B)** qPCR analyses of ND3, ND4L, CO1, CO2, CO3, ND2 and CYTB mRNA in WT and MT retina (n = 9-12). Data are shown as mean ± SEM. ***P* < 0.01, ****P* < 0.001 by 2-tailed unpaired Student's *t*-test.; Normality was assessed using the Shapiro-Wilk test, and equal variance was assessed using the F test.

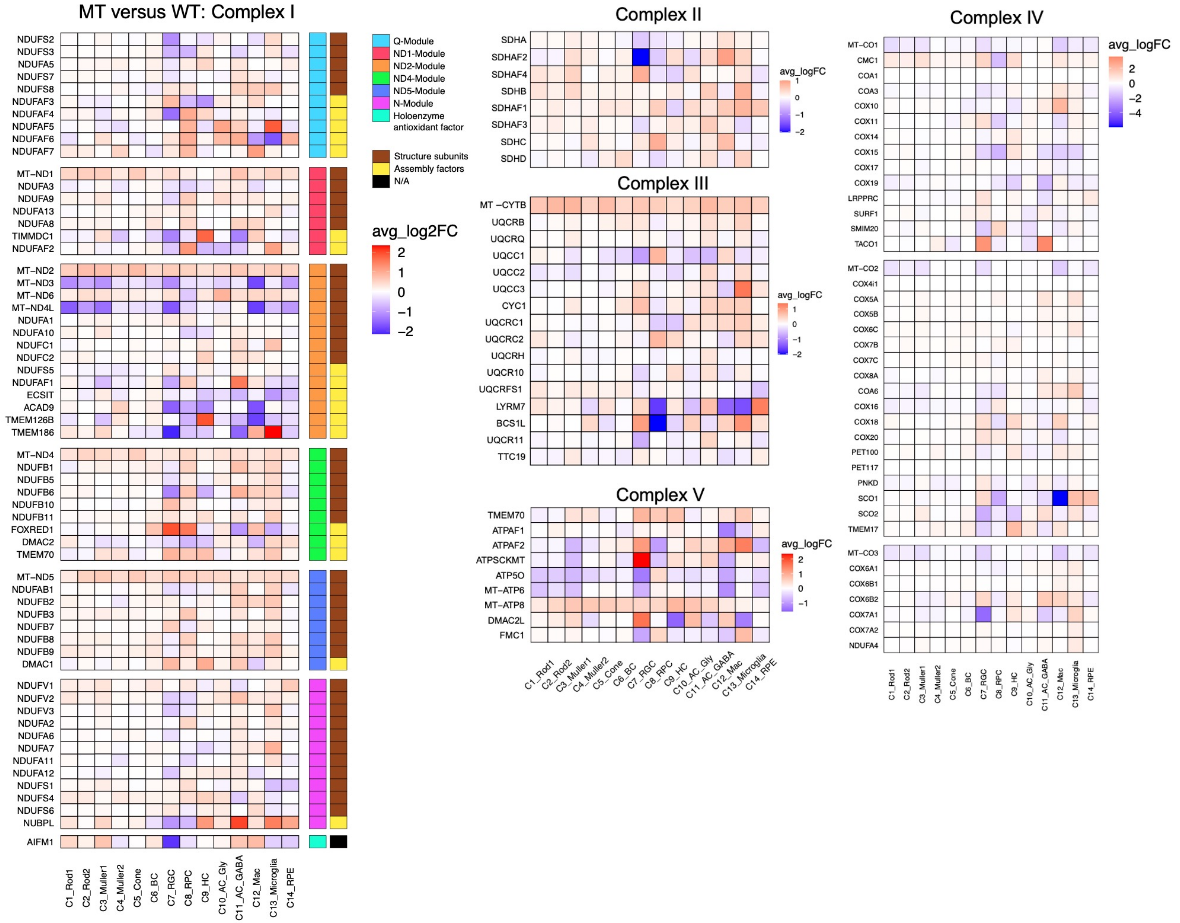

Supplemental Figure 3 (related to Figure 2): mitochondrial OXPHOS gene expression in retina cell clusters. Heatmaps displayed average log2 fold changes of expression for specific mitochondrial OXPHOS complex genes clustered into complex I, II, III, IV and V when comparing retinas of MT and WT mice. The specific OXPHOS modules of complex I were indicated.

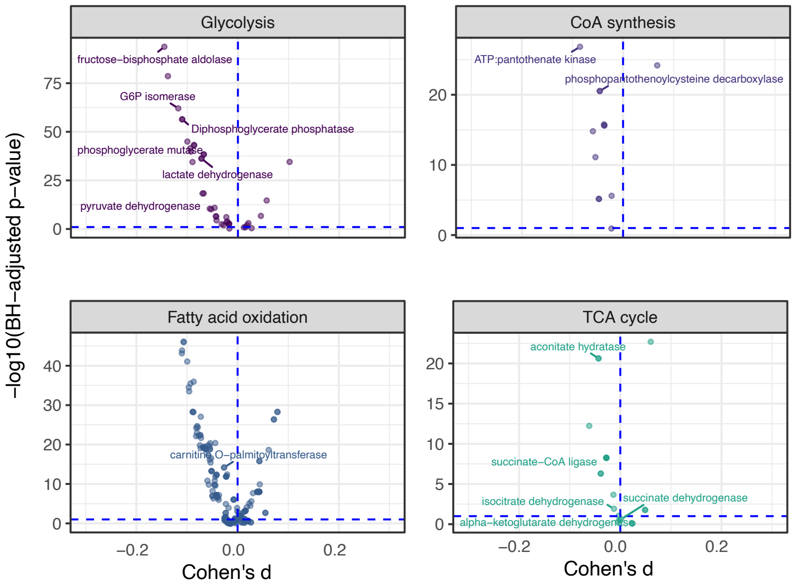

Supplemental Figure 4 (related to Figure 3): Mitochondrial metabolic pathway in mice retina. Compass-score differential activity test of mitochondrial and cytosolic bioenergetic pathways between MT and WT retina. Each dot represents a single biochemical reaction.

A

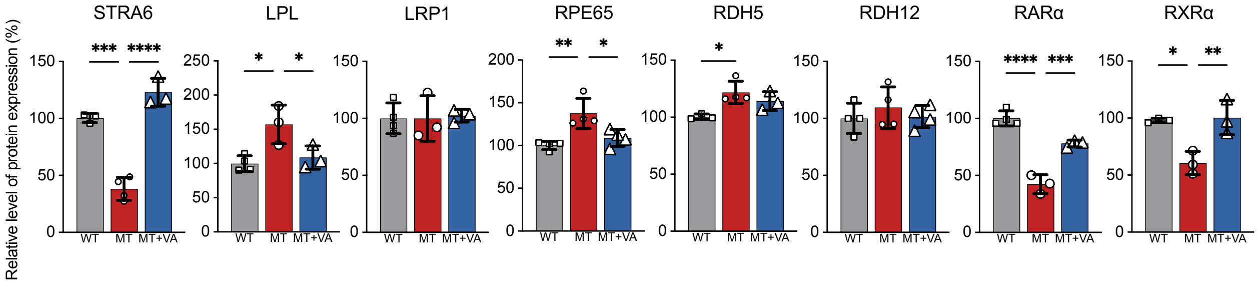

B

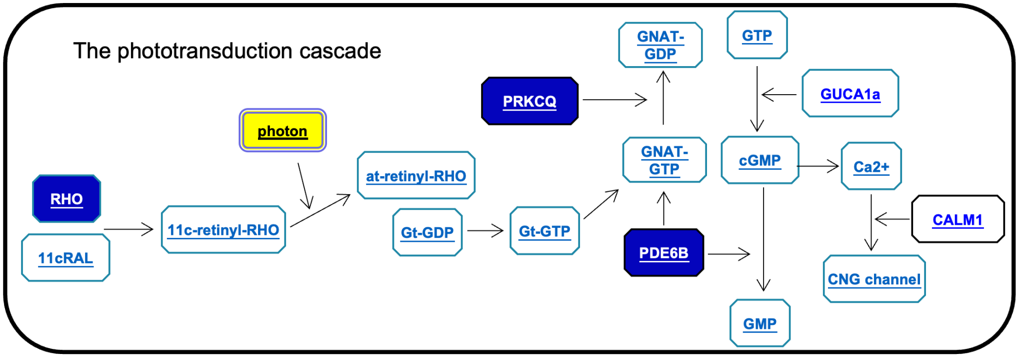

Supplemental Figure 5 (related to Figure 5): Restored vitamin A metabolism and signaling pathway. (A) Quantification of STRA6, LPL, LRP1, RPE65, RDH5, RDH12, RARα and RXRα in WT, MT, and MT+VA retina. Representative of 3 to 4 independent experiments. Data are shown as mean ± SEM. **P* < 0.05, ***P* < 0.01, ****P* < 0.001, *****P* < 0.0001 by 1-way ANOVA followed by Tukey post hoc test. **(B)** Schematic illustration of phototransduction pathway. The levels of of dark blue colored proteins was analyzed by western blotting in Figure 5, C and D.

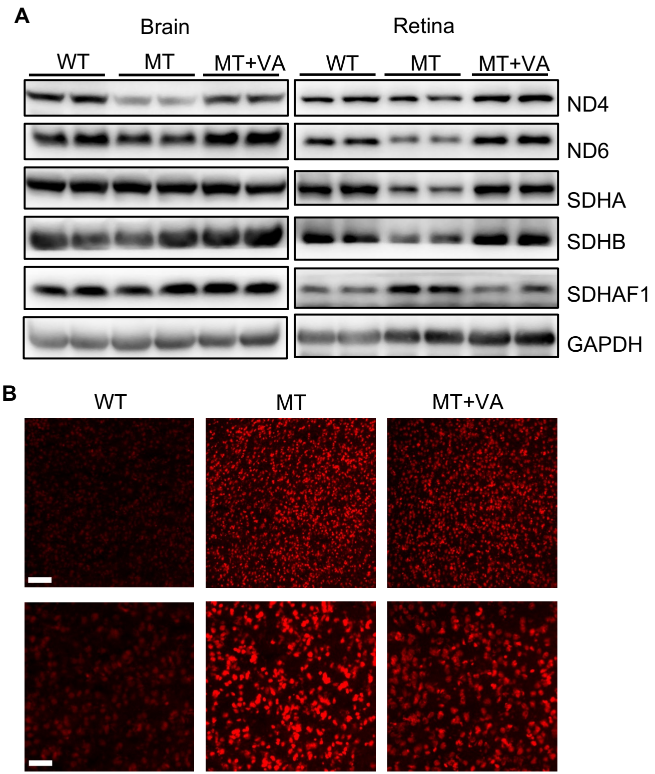

Supplemental Figure 6 (related to Figure 6): Restored OXPHOS translation and mitochondrial function in mutant brain and retina. (A) Western blot analysis of antioxidant proteins. Total cellular proteins in WT, MT and MT+VA brains and retinas were electrophoresed with PAGE and hybridized with ND4 and ND6 (subunits of complex I), SDHA and SDHB (subunits of complex II) and SDHAF1 (assemble factor of complex II) antibodies and GAPDH as a loading control, respectively. Representative of 3 to 4 independent experiments. **(B)** Assessment of ROS content by Dihydroethidium staining in the frozen-sections of brains of WT, MT, MT+VA mice. Scale bars: 50 μm (upper panel), 100 μm (lower panel).

Supplemental Table 1 (related to Figure 2): **Curated mitochondrial and bioenergetic gene list**

mtDNA GENE COMPLEMENT

The mtDNA codes for 7 of the 45 proteins of complex I (MT-ND1, MT-ND2, MT-ND3, MT-ND4, MT- ND4L, MT-ND5, MT-ND6), 1 of the 11 subunits of complex III (MT-CYB), 3 of the 13 subunits of complex IV (MT-CO1, MT-CO2, MT-CO3), and 2 of the approximately 17 subunits of complex V (MT-ATP6, MT-ATP8), mtDNA genes are highlighted in red below.

MITOCHONDRIAL OXPHOS Complex I

***Q-Module*:**
Structural Subunits: NDUFS2, NDUFS3, NDUFS7, NDUFS8, NDUFA5

Assembly Factors: NDUFAF3, NDUFAF4, NDUFAF5, NDUFAF6, NDUFAF7

***Q/ND1-Module*:**
Structural Subunits: MT-ND1, NDUFA3, NDUFA8, NDUFA9, NDUFA13

Assembly Factors: NDUFAF2, TIMMDC1

ND2-Module:

Structural Subunits: MT-ND2, MT-ND3, MT-ND6, MT-ND4L, NDUFA1, NDUFA10, NDUFC1, NDUFC2, NDUFS5

Assembly Factors: ACAD9, COA1, ECSIT, NDUFAF1, TMEM126B, TMEM186

ND4-Module:

Structural Subunits: MT-ND4, NDUFB1, NDUFB4, NDUFB5, NDUFB6, NDUFB10, NDUFB11

Assembly Factors: DMAC2 (ATP5SL), FOXRED1, TMEM70

ND5-Module:

Structural Subunits: MT-ND5, NDUFAB1, NDUFB2, NDUFB3, NDUFB7, NDUFB8, NDUFB9

Assembly Factors: DMAC1 (TMEM261)

N-Module:

Structural Subunits: NDUFA2, NDUFA6, NDUFA7, NDUFA11, NDUFA12, NDUFS1, NDUFS4, NDUFS6, NDUFV1, NDUFV2, NDUFV3

Assembly Factors: NUBPL

Holoenzyme Antioxidant factor: AIFM1

Complex II

Structural Subunits: SDHA, SDHB, SDHC, SDHD

Assembly Factors: SDHAF1, SDHAF2, SDHAF3, SDHAF4

Complex III

Structural Subunits: MT-CYB, UQCRB, UQCRQ, CYC1, UQCR10, UQCRC1, UQCRC2, UQCRFS1, UQCRH, UQCR11

Assembly Factors: UQCC1, UQCC2, UQCC3, LYRM7, BCS1L, TTC19

Complex IV

Structural Subunits: MT-COI, MT-CO2, COX4i1, COX5A, COX5B, COX6C, COX7B, COX7C, COX8A, MT-CO3, COX6A1, COX6B1, COX6B2, COX7A1, COX7A2, NDUFA4

Assembly Factors: CMC1, COA1, COA3, COX10, COX11, COX14, COX15, COX17, COX19, LRPPRC, SURF1, SMIM20, TACO1, COA6, COX16, COX18, COX20, PET100, PET117, PNKD, SCO1, SCO2, TMEM17

Complex V

Structural Subunits: ATP5F1A, ATP5F1B, ATP5F1C, ATP5F1D, ATP5F1E, ATP5MC1, ATP5MC2, ATP5MC3, ATP5ME, ATP5MG, ATP5MF, ATP5PB, ATP5PD, ATP5PF, ATP5PO, MT-ATP6, MT-ATP8, ATP5MJ, ATP5MK, DMAC2L, FMC1, TMEM70

Assembly Factors: ATP5IF1, ATPAF1, ATPAF2, ATPSCKMT

ANTIOXIDANT DENFENSES

APEX1, CAT, GPX1, GPX4, GSR, KEAP1, NFE2L2, PPX, SOD1, SOD2, TXN, TXN2, TRXR1, TRX2, TRX3

Supplemental Table 2 (related to Figure 3): Curated visual related pathway gene list

The canonical retinoid cycle in rods

RLBP1, RBP3, STRA6, TTR, RBP4, RHO, SDR9C7, HSD17B6, RDH16, DHRS9, RDH5, HSD17B1, RDH8, RPE65, MYO7A, RDH12, ABCA4, CYP4V2, RDH11, RDH10, RBP1, LRAT, NAPEPLD

The phototransduction cascade

RHO, NMT2, NMT1, GNAT1, SAG, FNTA, FNTB, GNGT1, GNB1, GUCY2D, GUCY2F, GUCA1B, GUCA1A, GUCA1C, GRK7, GRK4, GRK1, RCVRN, PPEF1, CNGB1, CNGA1, CALM1, PDE6G, PDE6A, PDE6B, GNB5, RGS9, RGS9BP, METAP1, METAP2, PRKCA, PRKCQ, CAMKMT, SLC24A1

Glutamate Neurotransmitter Release Pathway

STXBP1, CPLX1, RIMS1, SNAP25, RAB3A, SYT1, VAMP2, STX1A, TSPOAP1, UNC13B, PPFIA2, PPFIA4, PPFIA3, PPFIA1, GLS, GLS2, SLC38A2, SLC1A6, SLC1A2, SLC1A3, SLC1A1, SLC1A7, ARL6IP5, SLC17A7

GABA sythesis, release, reuptake and degradation

STXBP1, CPLX1, RIMS1, SNAP25, SLC32A1, RAB3A, SYT1, GAD2, GAD1, VAMP2, HSPA8, DNAJC5, STX1A, ALDH5A1, ABAT, SLC6A13, SLC6A11, SLC6A1, SLC6A12

Neurotansmitter uptake and metabolism in glia cells

GLUL, SLC1A3, SLC1A2, SLC38A1

Glutamate binding, activation of AMPA Receptor

CAMK2G, CAMK2D, CAMK2A, CAMK2B, GRIA1, GRIA3, GRIA4, EPB41L1, MDM2, DLG4, CACNG4, CACNG3, CACNG8, CACNG2, MYO6, DLG1, AKAP5, PRKCG, PRKCA, PRKCB, AP2S1, AP2B1, AP2A1, AP2A2, AP2M1, GRIA2, GRIP2, GRIP1, PICK1, NSF, TSPAN7

Activation of NMDA Receptor

ACTN2, NEFL, GRIN2B, GRIN1, GRIN2D, GRIN2A, GRIN2C, DLG4, DLG1, DLG2, DLG3, LRRC7, CAMK2G, CAMK2D, CAMK2B, CAMK2A, GRIA1, GRIA2, GRIA4, GRIA3, CALM1, RASGRF1, RASGRF2, KRAS, NRAS, HRAS, RPS6KA6, RPS6KA1, RPS6KA2, RPS6KA3, CREB1, MAPK3, MAPK1, PDPK1, NRGN, SRC, ERBB4, NRG1, PRKAR2A, PRKAR2B, PRKAR1A, PRKAR1B, PRKACA, PRKACB, PRKACG, ADCY8, ADCY1, PRKX, CAMKK2, PRKAA1, PRKAA2, PRKAB1, PRKAB2, PRKAG2, PRKAG1, PRKAG3,

TUBB6, TUBB2A, TUBB4B, TUBB1, TUBB3, TUBB2B, TUBB8, TUBB4A, TUBB8B, TUBA8, TUBA4B, TUBAL3, TUBA3E, TUBA1B, TUBA1A, TUBA1C, TUBA3C, TUBA3D, TUBA4A, MAPT, KPNA2, CAMK4, CAMKK1, CAMK1, GIT1, ARHGEF7, RAC1, GRIN3B, GRIN3A, CASK, APBA1, LIN7C, LIN7B, LIN7A, KIF17, NBEA, PPM1E, PPM1F

Activation of KA receptor

GRIK3, PLCB3, PLCB1, PLCB2, GNB2, GNB4, GNB5, GNB3, GNB1, GNG8, GNGT2, GNGT1, GNG7, GNG13, GNG3, GNG10, GNG11, GNG4, GNG5, GNG12, GNG2, GRIK2, GRIK1, GRIK4, DLG4, DLG3, NCALD, CALM1, DLG1, GRIK5

GABA receptor activation

GABRG3, GABRG2, GABRB3, GABRB1, GABRB2, GABRA1, GABRA2, ARHGEF9, GABRA6, GABRA3, GABRA4, GABRA5, GABRQ, GABRR1, GABRR3, GABRR2, NPTN, GABBR1, GABBR2, KCNJ10, KCNJ4, KCNJ12, KCNJ16, KCNJ2, KCNJ3, KCNJ5, KCNJ6, KCNJ9, KCNJ15, GNB2, GNB4, GNB5, GNB3, GNB1, GNG8, GNGT2, GNGT1, GNG7, GNG13, GNG3, GNG10, GNG11, GNG4, GNG5, GNG12, GNG2, GNAI3, GNAI1, GNAI2, GNAT3, ADCY7, ADCY6, ADCY2, ADCY9, ADCY4, ADCY8, ADCY3, ADCY5, ADCY1, GNAL

Supplemental Table 3 (related to Figure 4): **Summary of fundus lesion frequency in 2-mo-old mouse of different group**

group	n	LE (n)	LE rate	RE (n)	RE rate	BE (n)	BE rate	Total rate
WT	14	0	0	0	0	0	0	0
MT	9	3	33.33%	3	33.33%	1	11.11%	77.80%
MT+VA	8	3	37.50%	3	37.50%	0	0	75%

LE: left eye only; RE: right eye only; BE: both eye

Supplementary Table 4. Nucleotide sequences of the primers used for qRT-PCR

Mouse Gene	Genbank Accession number	Forward Primer (5' to 3')	Reverse Primer (5' to 3')	Amplicon size (bp)	Annealing temperatures (℃)
ND3	NC_005089.1 (9459-9806)	ATTAACCTGTA CACTGTTATCT	TTACTCTGTTCA TTCTAATCCT	348	60
ND4L	NC_005089.1 (9877-10173)	ATGCCATCTAC CTTCTTCAACC	TTAGCATTGTA GTAGGTTGAGA	297	60
CO1	NC_005089.1 (5328-6872)	ATGTTCATTAAT CGTTGATTAT	ATTTATTCGTGG GAATGCTATAT	294	60
CO2	NC_005089.1 (7013-7696)	TACCCATTCCA ACTTGGTCTA	CTTCAGTATCAT TGGTGCCCTA	314	60
CO3	NC_005089.1 (8607-9390)	ACTTTACTTCAC CATCCTCCA	AAGATCCTCAT CAATAAATGGA	275	60
ND2	NC_005089.1 (3914-4951)	ATAAATCCTAT CACCCTTGCC	ATGTTCCTAGTT GTTTATAGT	250	60
CYTB	NC_005089.1 (14145-15288)	TACGAAAAACA CACCCATTAT	TCAGGTTTCTAT AAATGTATAT	329	60
β-actin	NM_007393.5	GGCTGTATTCC CCTCCATCG	CCAGTTGGTAA CAATGCCATGT	154	60

Primer specificity was confirmed by blasting the primer sequences against the NCBI database.

Supplemental Table 5. The information of the applied primary and secondary antibodies

Antibodies	SOURCE	IDENTIFIER	Application
Mouse monoclonal anti-GAPDH	Proteintech	Cat#: 60004-1-Ig	WB
Rabbit anti-STRA6	Proteintech	Cat#: 22001-1-AP	WB
Rabbit anti-RPE65	ABclonal	Cat#: A9615	WB
Rabbit anti-RDH5	Abcam	Cat#: ab200197	WB
Rabbit anti-LRP1	ABclonal	Cat#: A0633	WB
Rabbit anti-LPL	ABclonal	Cat#: A4115	WB
Rabbit anti-RARα	ABclonal	Cat#: A19551	WB
Rabbit anti-RXRα	ABclonal	Cat#: A19105	WB
Rabbit anti-RDH12	Proteintech	Cat#: 13289-3-AP	WB
Rabbit monoclonal anti-RHO	ABclonal	Cat#: A7245	WB
Rabbit anti-PKC alpha	ABclonal	Cat#: A0267	WB
Rabbit anti-Vimentin	ABclonal	Cat#: A11952	WB
Rabbit anti-PRKCQ	ABclonal	Cat#: A5834	WB
Rabbit anti-PDE6B	ABclonal	Cat#: A6942	WB
Rabbit anti-NMDAR1	ABclonal	Cat#: A7677	WB
Rabbit anti-GNB3	ABclonal	Cat#: A1387	WB
Rabbit anti-GABBR1	ABclonal	Cat#: A10504	WB
Rabbit anti-GluR1	ABclonal	Cat#: A1826	WB
Rabbit monoclonal anti-VAMP2	ABclonal	Cat#: A4235	WB
Rabbit anti-SOD1	Proteintech	Cat#: 10269-1-AP	WB
Rabbit anti-SOD2	ABclonal	Cat#: A19576	WB
Rabbit anti-Catalase	ABclonal	Cat#: A11780	WB
Rabbit anti-SDHA	Proteintech	Cat#: 14865-1-AP	WB
Rabbit anti-SDHB	Proteintech	Cat#:10620-1-AP	WB
Rabbit anti-SDHAF1	Abcam	Cat#: ab185222	WB
Rabbit anti-ND4	Proteintech	Cat#: 26736-1-AP	WB
Rabbit anti-ND6	ABclonal	Cat#: A17991	WB
Anti-Mouse IgG	Beyotime	Cat#: A0216	WB
Anti-Rabbit IgG	Beyotime	Cat#: A0208	WB
Mouse anti-Brn3a	Sigma-Aldrich	Cat#: MAB1585	IHC-Fr
Rabbit monoclonal anti-Vimentin	ABclonal	Cat#: A19607	IHC-Fr
Mouse anti-Rhodopsin	Abcam	Cat#: ab98887	IHC-Fr
YSFluor™ 488 Anti-Rabbit	YESAON	Cat#: 33106ES60	IHC-Fr
YSFluor™ 594 Anti-Mouse	YESAON	Cat#: 34112ES60	IHC-Fr
