# Supplementary material for: Vitamin A treatment restores vision failures arising from Leber’s hereditary optic neuropathy–linked mtDNA mutation
Source: JCI Insight. 2025 Mar 4;10(8):e188962. doi: 10.1172/jci.insight.188962 (PMC12038914; doi:10.1172/jci.insight.188962)

Full unedited gel for Figure 3E

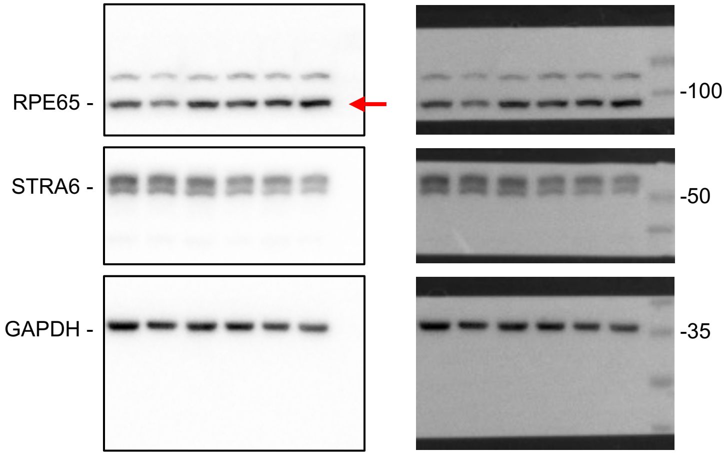

Full unedited gel for Figure 5A

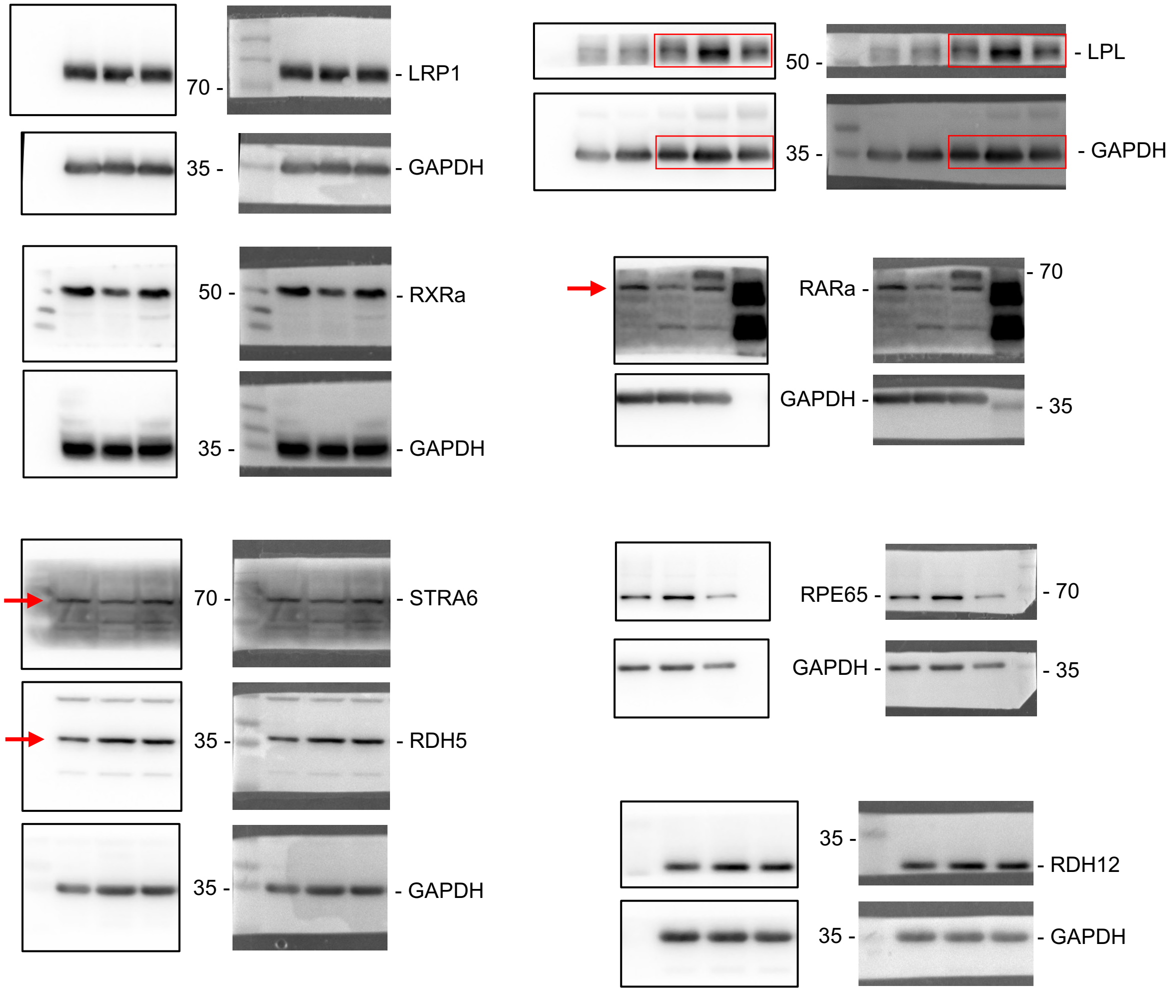

Full unedited gel for Figure 5C

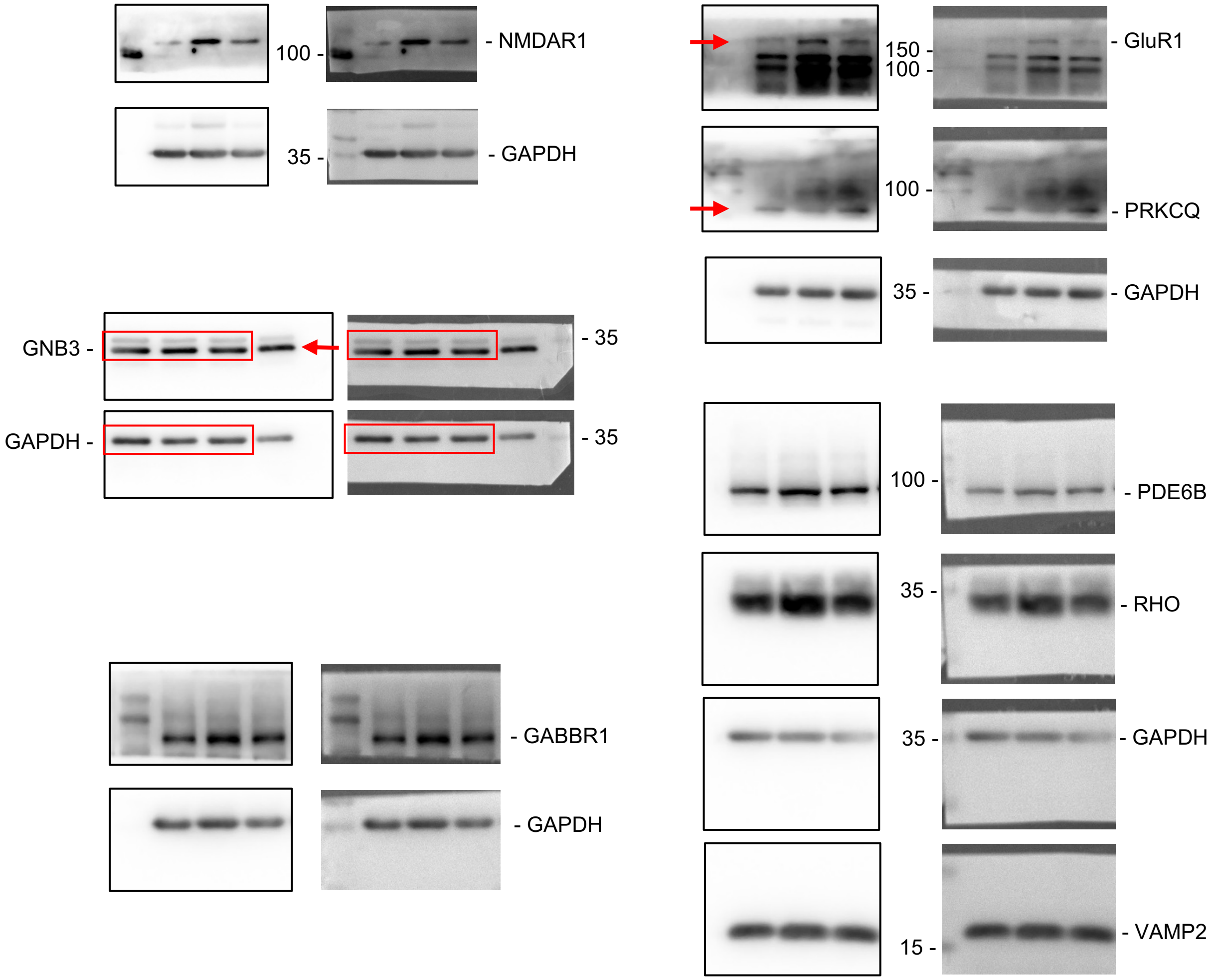

Full unedited gel for Figure 6B

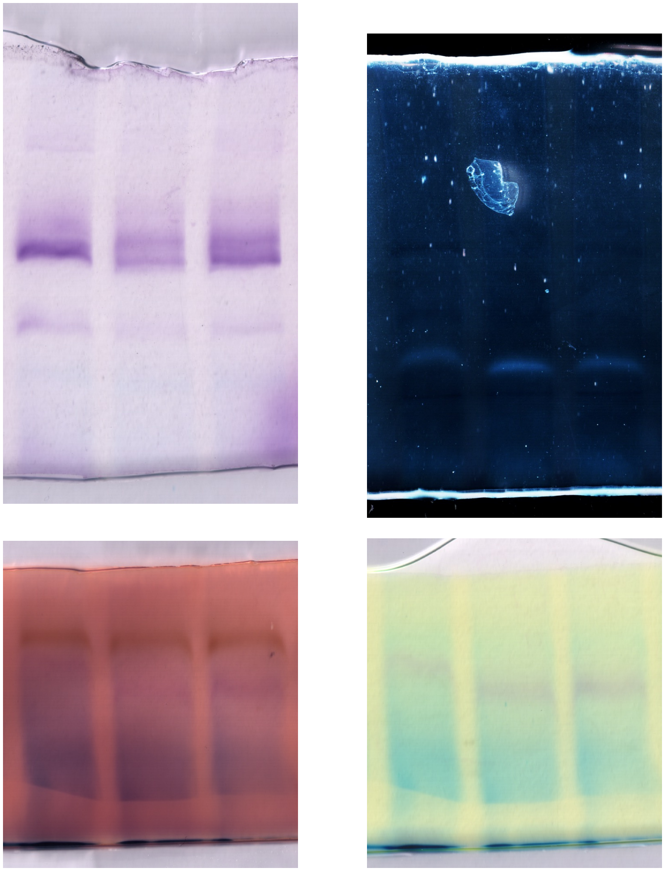

Full unedited gel for Figure 6F

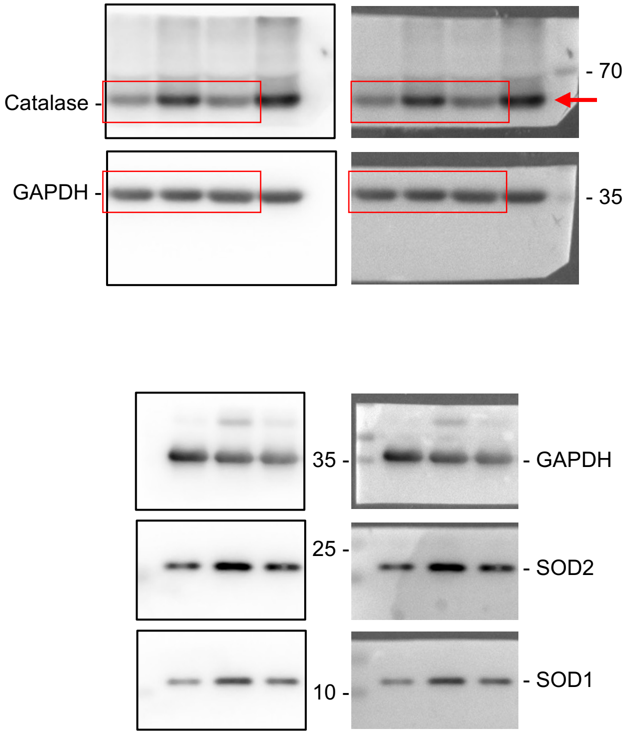

Full unedited gel for Supplemental Figure 6A

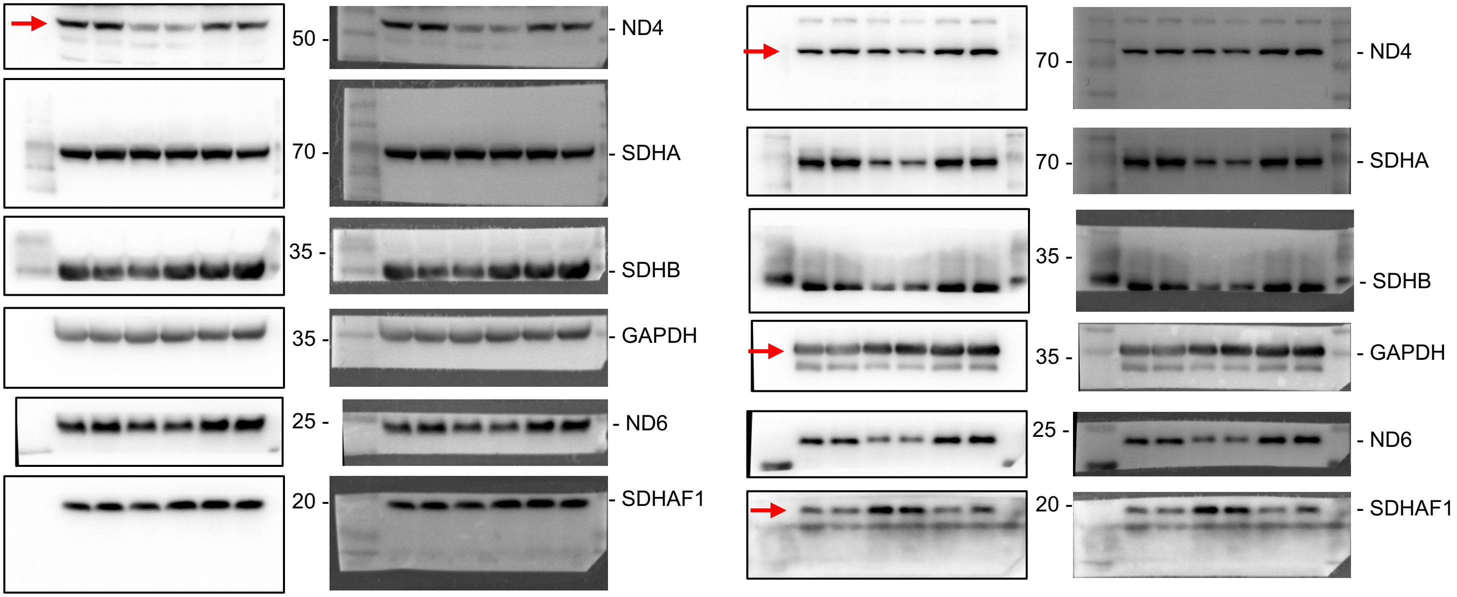

Supplement: Unedited blot and gel images [file jciinsight-10-188962-s020.pdf]
